# Rigorous sampling of docking poses unveils binding hypothesis for the halogenated ligands of L-type Amino acid Transporter 1 (LAT1)

**DOI:** 10.1038/s41598-019-51455-8

**Published:** 2019-10-21

**Authors:** Natesh Singh, Bruno O. Villoutreix, Gerhard F. Ecker

**Affiliations:** 1 University of Lille, Inserm, Institut Pasteur de Lille, U1177 - Drugs and Molecules for living Systems, F-59000 Lille, France; 2 Department of Pharmaceutical Chemistry, University of Vienna, Althanstrasse 14, 1090 Wien, Austria

**Keywords:** Computational models, Protein structure predictions

## Abstract

L-type Amino acid Transporter 1 (LAT1) plays a significant role in the growth and propagation of cancer cells by facilitating the cross-membrane transport of essential nutrients, and is an attractive drug target. Several halogen-containing L-phenylalanine-based ligands display high affinity and high selectivity for LAT1; nonetheless, their molecular mechanism of binding remains unclear. In this study, a combined in silico strategy consisting of homology modeling, molecular docking, and Quantum Mechanics-Molecular Mechanics (QM-MM) simulation was applied to elucidate the molecular basis of ligand binding in LAT1. First, a homology model of LAT1 based on the atomic structure of a prokaryotic homolog was constructed. Docking studies using a set of halogenated ligands allowed for deriving a binding hypothesis. Selected docking poses were subjected to QM-MM calculations to investigate the halogen interactions. Collectively, the results highlight the dual nature of the ligand-protein binding mode characterized by backbone hydrogen bond interactions of the amino acid moiety of the ligands and residues I63, S66, G67, F252, G255, as well as hydrophobic interactions of the ligand’s side chains with residues I139, I140, F252, G255, F402, W405. QM-MM optimizations indicated that the electrostatic interactions involving halogens contribute to the binding free energy. Importantly, our results are in good agreement with the recently unraveled cryo-Electron Microscopy structures of LAT1.

## Introduction

The availability of experimentally determined high-resolution protein-ligand structures intensely expedites the design and optimization of small molecules during the drug discovery process through elaborative multiparameter ligand optimization. The information acquired from the ligand-protein complex indeed offers a rational basis to improve the potency and selectivity of the ligand^1^. Also, this knowledge can assist medicinal chemists in optimizing the pharmacokinetic properties of the ligand, without disrupting the important protein-ligand contacts^1,2^. Additionally, it facilitates the elucidation of molecular features involved in ligand-binding, leading, for example, to agonist and antagonist actions of the small molecules^3^. However, structural and molecular interpretation of a protein-ligand complex itself is an arduous task and has been unsuccessful for various significant drug targets^4^. In the absence of an atomic structure of a protein, researchers can employ different methods to investigate the binding of small molecules and explore novel lead compounds. The most prominent and popular approach combines the use of homology modeling and molecular docking^5^. However, the sequence similarity between target and template proteins plays a crucial role in determining the success of homology modeling and subsequent docking studies^6^. As the sequence identity and similarity between the target and the 3D templates decrease, the structural model may become less accurate, thereby reducing the chances of defining precise binding modes^7^. However, docking procedures have been shown in numerous cases to be capable of generating the correct binding orientation by adequately exploring the conformational space within the binding site^8–10^.

The L-type Amino acid Transporter (LAT1, SLC7A5) represents a promising drug target responsible for nutrient uptake, brain drug delivery, and tumor growth^11,12^. LAT1 is a Na^+^-independent amino acid exchanger with broad substrate specificity toward large hydrophobic/aromatic amino acids such as leucine, tyrosine, and tryptophan^13^. LAT1 is highly expressed in the Blood-Brain Barrier (BBB)^14^, placenta^15^, immune T cells^16,17^, and numerous types of cancers^13,18,19^. For these reasons, LAT1 is intensively studied for cancer therapeutics and diagnostics^20–22^, treatment of neurological disorders such as Parkinson’s disease^23^ and identification of compounds that can cross the BBB^24^. At the BBB, LAT1 allows passage of several clinical drugs that mimic amino acids such as thyroid hormones (T_3_, T_4_)^25^, L-DOPA^26^, melphalan^27,28^, baclofen^29^, and gabapentin^27^. LAT1 also stimulates mammalian Target of Rapamycin (mTOR) activity by mediating uptake of leucine in cells^30^. The topology of LAT1 can be described by assemblies of 12 transmembrane helices (TMs) arranged in a 5 + 5 two-fold inverted repeats^31^, and it is covalently bound to the glycoprotein CD98hc (4F2hc, or SLC3A2) via a disulfide bridge between C164 of LAT1 and C210 of CD98hc^32^. It has been demonstrated that LAT1 is the sole transport-competent unit, while CD98hc does not play any specific role in the intrinsic transport function^33^. CD98hc instead functions as a molecular chaperone enabling plasma membrane localization and stabilization of LAT1^34^.

**Figure 1:**
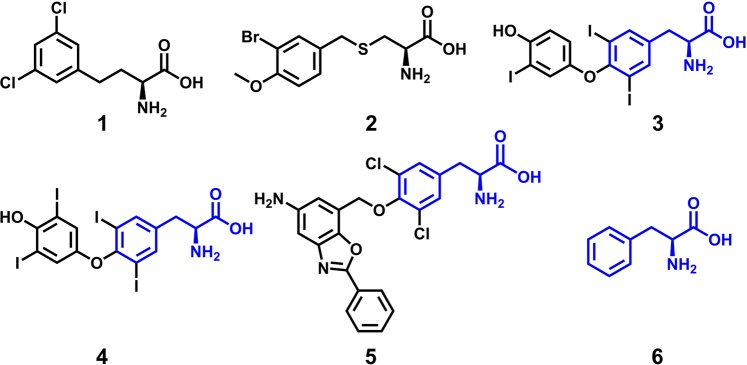
Chemical structures of LAT1 inhibitors. **1**, L-2-amino-4-(3,5-dichlorophenyl)butanoic acid (pIC_50_: 6.19) **2**, S-(3-bromo-4-methoxybenzyl)-L-cysteine (pIC_50_: 4.48) **3**, L-triiodothyronine (L-T_3_) (pIC_50_: 5.23) **4**, L-tetraiodothyronine (L-T_4_) (pIC_50_: 4.04) **5**, (S)-2-amino-3-(4-((5-amino-2-phenylbenzo[d]oxazol-7-yl)methoxy)-3,5-dichlorophenyl)propanoic acid (KYT-0353) (pIC_50_: 7.22) **6**, L-phenylalanine (pIC_50_: 4.96).

Despite the immense biological significance of LAT1, our understanding of the molecular features that govern ligand binding is limited. Past studies have highlighted the potential of homology modeling to be used for understanding the transport mechanism^33,35^, structure-function relationship^36^, and discovery of novel ligands of LAT1^37^. Recently we reported potent halogen-containing inhibitors **1**-**2** (Fig. 1) of LAT1 through dynamic pharmacophore-based screening of small molecule libraries, raising the question of whether the halogens potentiate the binding through non-covalent interactions^38^. Given the widespread occurrence of the halogens (X) in several high-affinity LAT1 ligands such as L-T_3_ (X = I) **3**^27,39,40^, L-T_4_ (X = I) **4**^27,39^, and KYT-0353 (X = Cl) **5**^21,41^ (Fig. 1) and their incorporation in rational drug design, it is possible that the halogens not only modulate the lipophilicity but also participate in polar interactions, thus driving the binding affinity. Halogen substituents are amphipathic and are capable of serving as both halogen bond (X-bond) donors to carboxyl oxygen, carbonyl oxygen or aromatic π-system in the direction of the electropositive crown (σ-hole) and hydrogen bond or X-bond acceptors in the perpendicular direction^42,43^. In an X-bond interaction, the σ-hole on the halogens (I, Br, and Cl) functions as a Lewis acid to attract a variety of electron-rich Lewis bases (O, N, and S) in an analogous fashion to classical hydrogen bond interactions^44^. The presence of other substituents (e.g., electron-withdrawing or electron-donating groups) affects the polarization of halogens, and consequently the strength of the X-bond interaction. The X-bond donor strength decreases in the order I > Br > Cl > F^45^.

The compounds **3**–**5** share a common substructure “2-Amino-3-phenylpropanoic acid” (L-phenylalanine) **6**^28^ (Fig. 1, depicted in blue) that is a system L substrate and a widely used chemical tool to evaluate the transport activity of LAT1^27^. Over the past three decades, several phenylalanine derivatives have been synthesized and tested for LAT1 activity that led to a highly distinguished Structure-Activity Relationship (SAR) of this prototype ligand^27,40,46–50^. Interestingly, the phenylalanine-based ligands exhibit features of both “continuous” and “discontinuous” SARs^51^, depending on the corresponding substitution sites over the scaffold. A continuous SAR is characterized by a smooth activity hypersurface, where a clear trend in biological activity could be observed upon systematic chemical changes, while a discontinuous SAR, in contrast, is described by a rugged landscape where minor structural modifications lead to drastic potency differences.

In this study, we combine homology modeling, molecular docking, and Quantum Mechanics-Molecular Mechanics (QM-MM) to define a binding hypothesis for the halogenated phenylalanine-based ligands of LAT1. In the first step, compounds were docked to a homology model of human LAT1, which was followed by the assessment of the docking poses using a common scaffold clustering technique to identify a common binding mode. Subsequently, selected docking poses were optimized through hybrid QM-MM method to calculate the interaction energy of the halogen-bonded adducts. Finally, the results from the docking studies were compared with the newly solved cryo-Electron Microscopy (cryo-EM) structures (PDB IDs: 6IRT, 6JMQ) of human LAT1-CD98 complex^12,32^.

## Results

### Structure-Activity relationships (SAR) of phenylalanine-based ligands of LAT1

**Figure 2:**
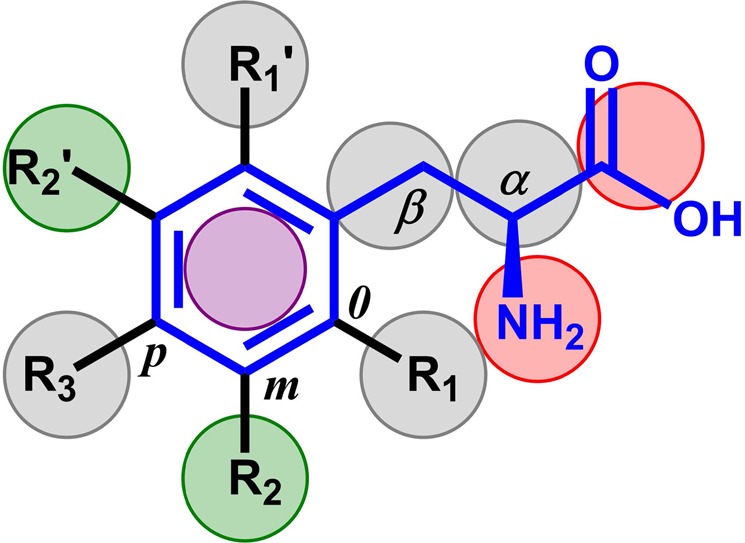
2D representation of the L-phenylalanine scaffold depicting the SAR. The α-amino and the α-carboxyl groups are indicated by red spheres, and both the positions are sterically restrained. The aliphatic portion of the side chain is marked as α and β (grey spheres), and both the sites can tolerate modifications. The aromatic ring of the side chain is indicated by a violet sphere. The different positions on the aromatic ring are as follows: ortho (*o*) (R_1_ and R_1_’, grey spheres), meta (*m*) (R_2_ and R_2_’, green spheres) and para (*p*) (R_3_, grey sphere). All positions on the aromatic ring can tolerate modifications and steric bulk. However, the meta positions (green spheres) have been shown critical for affinity.

The studies so far performed on LAT1 have revealed a number of interesting findings regarding the recognition and binding specificity of the compounds. On the basis of available SAR data, we deduced a model of the prototype ligand L-phenylalanine **6** (Figs 1 and 2; Table 1) that shows a discontinuous SAR with activity cliffs at the substitution positions α-carboxyl, and α-amino, while at the positions α, β, R_1_, R_2,_ and R_3_ a continuous SAR is observed. The most crucial structural feature of the active LAT1 ligands is their amino acid moieties, and their alteration or replacement generally leads to inactive compounds or a dramatic loss of potency (Table 1). In particular, the α-amino group has been found to be more critical than the α-carboxyl group to attain efficient binding. For example, the modification of the α-carboxylic group to a hydroxamic acid moiety **7**, methylsulfonamide **8**, tetrazole **9**, hydroxyl **10**, sulfonic acid **11** or carboxylic ester **12** results in the sharp decrease in activity^27,46,49,52^, while substitutions on the α-amino group **13**, **14** leads to reduced activity or a total loss of activity (Table 1)^27,38,52,53^. Thus, it can be inferred that substituents at these positions that increase the van der Waals volume are not favorable for the binding possibly due to steric hindrance in the binding site. The transfer of the α-amino group to the β position **15**, **16** leads to reduced activity, indicating that the optimal position of the amino group should be at Cα^38,47^. This might indicate a decrease of ionic interactions of the amino group when it is moved from α to β position, resulting in reduced activity. The replacement of the Cα hydrogen with methyl **17**, **18** has been reported to be tolerable though with a reduced affinity^27,47^. The meta position of the aromatic ring has been described to be most favorable for affinity, as the substituents are believed to interact with a secondary hydrophobic sub-pocket in LAT1^38^. The ortho-substituted derivatives have not been evaluated extensively as compared to the meta or para ones. Nevertheless, several studies have confirmed the affinity trend meta > para^24,28,38,48^ (e.g., **19**, **20**). Interestingly, a few studies using methyl^48^ or fluoroethyl^54^ substituents have shown that inhibition follows the trend ortho **21** > para **22** > meta **23** and ortho **24** > meta **25** > para **26**, respectively. These conflicting results might indicate that LAT1 has specific steric and electronic requirements that require further investigation. Moreover, previous studies have shown that cyclization of the aliphatic side chain into cyclohexane **27**, cyclopentane **28**, or bicycloheptane **29** led to bioactive molecules, indicating that conformational restriction at the Cα/β position is possible^28,38,40^. Lastly, it may be inferred that LAT1 represents a case of heterogeneous SAR that is characterized by concurrent existence of different continuous and discontinuous SAR, where SAR continuity is observed within the limits of a structural constraint, i.e., amino acid moiety of the ligands.

**Table 1:** The Structure-Activity Relationships (SAR) of phenylalanine-related compounds of LAT1.


Cpd.	A	B	C	R	Biological Activity	Ref.
6	−CO_2_H	−NH_2_	H	PhCH_2_−	^a^85 ± 0.6	^49^
7	−CONHOH	−NH_2_	H	PhCH_2_−	^a^24 ± 0.5	^49^
8	−CONHSO_2_Me	−NH_2_	H	PhCH_2_−	^a^15 ± 0.7	^49^
9		−NH_2_	H	PhCH_2_−	^a^11 ± 1	^49^
10	−CH_2_OH	−NH_2_	H	PhCH_2_−	^a^23 ± 3	^46^
11	−SO_3_H	−NH_2_	H	PhCH_2_−	^a^21 ± 7	^46^
12	−CO_2_Me	−NH_2_	H	PhCH_2_−	^b^inactive	^27^
13	−CO_2_H	−NHMe	H	PhCH_2_−	^b^inactive	^27^
14	−CO_2_H		H	*p*-HOPhCH_2_−	^a^31 ± 1	^46^
15	−CO_2_H	H	H		^a^36 ± 7	^47^
16	−CO_2_H	H	H		^a^32 ± 2	^38^
17	−CO_2_H	−NH_2_	CH_3_	PhCH_2_−	^b^active	^27^
18	−CO_2_H	−NH_2_	CH_3_	*p*-HOPhCH_2_−	^a^38 ± 5	^46^
19	−CO_2_H	−NH_2_	H		^a^93 ± 2	^46^
20	−CO_2_H	−NH_2_	H		^a^64 ± 4	^46^
21	−CO_2_H	−NH_2_	H	*o*-MePhCH_2_−	^a^98	^48^
22	−CO_2_H	−NH_2_	H	*p*-MePhCH_2_−	^a^86	^48^
23	−CO_2_H	−NH_2_	H	*m*-MePhCH_2_−	^a^73	^48^
24	−CO_2_H	−NH_2_	H	*o*-FEtPhCH_2_−	^c^16.09 ± 3.74	^54^
25	−CO_2_H	−NH_2_	H	*m*-FEtPhCH_2_−	^c^48.95 ± 4.92	^54^
26	−CO_2_H	−NH_2_	H	*p*-FEtPhCH_2_−	^c^85.55 ± 4.40	^54^
27	−CO_2_H	−NH_2_	—		^c^7.7 ± 0.8	^28^
28	−CO_2_H	−NH_2_	—		^c^12.5 ± 1.1	^28^
29	−CO_2_H	−NH_2_	—		^c^26 ± 1	^28^

^a^Percent (%) inhibition.

^b^Bioactivity data was not reported.

^c^K_i_ (μM).

### Homology model of LAT1

The outward-occluded conformation of the arginine-agmatine antiporter (AdiC) with Protein Data Bank (PDB) entry 3L1L was used as a template for the homology modeling of human LAT1. AdiC shares a sequence identity of ∼20% and sequence similarity of ∼40% with the human LAT1. Notwithstanding the overall low sequence identity, the identity of amino acids in the substrate binding site of LAT1 is a crucial factor in ascertaining the success of docking studies against the putative homology model. Notably, the sequence alignment revealed a high identity of ∼40% and similarity of 68% for the residues within the substrate binding site, indicating a highly conserved binding environment in the two homologous transporters. As such, it seems reasonable to investigate the transporter-ligand interactions using a homology model. The models were constructed using MODELLER as described previously^38^ and were assessed on the basis of normalized Discrete Optimized Protein Energy (DOPE) score^55,56^. The top-ranked model in terms of the DOPE score (−0.41) was analyzed by means of residue hydrophobicity, and electrostatic potential calculations (see Supplementary Information, Figs S1, S2). In addition, the overall quality of the model was assessed using a Ramachandran plot (Fig. S3). The model had 91.7%, 6.0%, 1.1%, and 1.1% of the residues, respectively, assigned as the “most favored”, “additionally allowed”, “generously allowed”, and “disallowed” regions. The LAT1 model was also evaluated by measuring the Root Mean Square Deviation (RMSD) between its backbone atoms and those of the AdiC template structure. The RMSD (0.62 Å) is very low indicating further that the amino acids of LAT1 can fully accommodate in the template 3D structure (Fig. S4). Overall, the structural analysis strongly suggests that the homology model of LAT1 can be used for the docking studies.

### Binding pocket comparison between outward-occluded model and inward-open structure of LAT1

**Figure 3:**
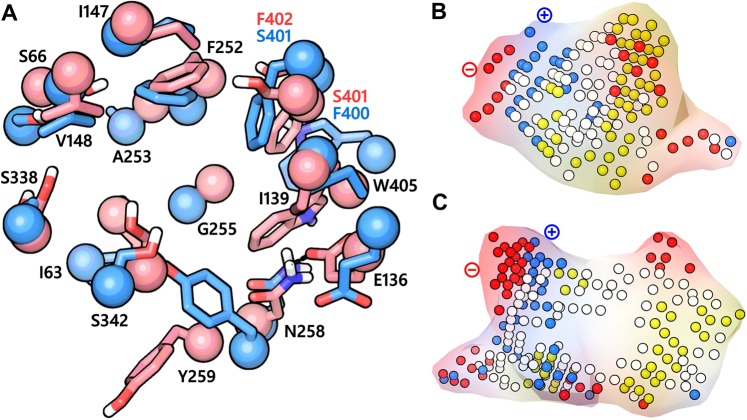
(**A**) The binding site of LAT1 in the outward-occluded conformation (red) superposed to the binding site of LAT1 in the inward-open state (PDB ID: 6IRT) (blue). The Cα atoms and the side chains are shown in space-filling and stick style, respectively. N258 and E136 in the outward-occluded model are engaged in a hydrogen bond interaction (black dotted line), possibly indicating a closed distal gate. While the side chain of E136 is oriented away from the binding site in the inward-open structure, suggesting an open distal gate permitting substrate access to the binding site from the cytoplasm. (**B**,**C)** are the negative images of the substrate binding cavity of the outward-occluded and the inward-open structure of LAT1, where the red, blue, yellow and white spheres are the site points corresponding to the hydrogen bond acceptor, hydrogen bond donor, hydrophobic and mixed character regions, respectively. The positive and the negative pole of the binding site are marked.

At the time of writing this manuscript, the structure of LAT1 bound to the classical system L inhibitor ‘2-Amino-2-norbornanecarboxylic acid’ (BCH) in the inward-open conformation (PDB ID: 6IRT)^32^ and in the inward-facing apo state (PDB ID: 6JMQ)^12^ were resolved with cryo-EM. These data allowed us to compare the substrate binding site of LAT1 in two distinct conformational states: outward-occluded and inward-open. The structure-based alignment of the two pockets displayed a relatively large Cα RMSD of 2.14 Å (Fig. 3A) highlighting the topological differences between the sites. Yet, the alignment revealed that with a few exceptions, nearly all the residues comprising the binding site in the outward-occluded model are identical to the inward-open structure suggesting further that our homology model is accurate. S401 and F402 (TM10) of the outward-occluded model corresponds to F400 and S401 of the inward-open structure. Importantly, the orientation of the sidechain of gate residue F252 in the inward-open structure is similar to the outward-occluded structure (Fig. 3A), while in the outward-open model this residue is oriented away from the pocket^50^. Also, the alignment showed an inter-residue hydrogen bond interaction in the outward-occluded model involving E136 (TM3) and N258 (TM6) (Fig. 3A). E136 and N258 of LAT1 are equivalent to the distal gate residues Y93 and E208 of AdiC that are also involved in hydrogen bond interactions^57^. In the inward-open structure, the side chain of E136 is oriented away from the pocket corresponding probably to an open gate-like condition (Fig. 3A). We hypothesize that E136 might be playing an important role in the translocation of the substrates, by opening and closing the distal gate through participation in hydrogen bond interaction, during an alternating-access cycle. Next, we analyzed and compared the binding site properties of the two structures using SiteMap v3.4^58^. This tool analyzes the binding pockets by using grid points, called site points, and then employs the van der Waals (vdW) and electrostatic interactions of a probe positioned at each point to create field maps. The probe simulates a water molecule with a vdW radius of 1.6 Å. SiteMap partitions the solvent accessible surface into three types of regions: hydrophobic, hydrophilic, and mixed character regions. The hydrophilic region is further divided into hydrogen bond donor, hydrogen bond acceptor, and metal-binding regions. The hydrogen bond donor and acceptor properties indicate the degree to which a ligand might be expected to donate and accept hydrogen bonds, respectively^58^. The outward-occluded model yielded a SiteScore of 1.184; a volume of ∼206 Å^3^ and a total solvent accessible surface area of 377.76 Å^2^. The negative image of the binding pocket consists of 25% hydrophobic region (yellow spheres); 57% hydrophilic region (red and blue spheres) and 18% mixed character region (white spheres) (Fig. 3B). The hydrophilic zone is subdivided into hydrogen bond donor and acceptor regions. The hydrogen bond donor region consists of 67% of the hydrophilic region and the hydrogen bond acceptor region, 33% of the hydrophilic region. The hydrogen bond acceptor region refers to the degree that a well-structured ligand could interact with hydrogen bond donor residues. The hydrophobic region contains residues like I63, I64, G65, I139, I140, V148, F252, A253, G255, G256, Y259, C335, V339, L349, F402, W405, and V408, whereas the hydrophilic region contains residues like T62, S66, G67, D116, E136, S143, S144, N258, S338, S342, T345, and S401. The binding pocket of the inward-open structure returned a SiteScore of 1.134 and has a volume of ∼523 Å^3^ and a total solvent accessible surface of 965.89 Å^2^. The pocket is large and elongated in shape and has less sphericity compared to the outward-occluded pocket. The binding pocket of the inward-open structure is ∼12% hydrophobic, ∼65% hydrophilic, and ∼23% mixed (hydrophobic and hydrophilic) (Fig. 3C). The hydrophobic region contains residues I58, G61, I63, I64, G65, I68, I140, V148, F200, A201, L251, F252, A253, Y254, G255, G256, W257, Y259, L260, Y289, G337, G341, F344, F400, W405, and C407. The hydrophilic region is subdivided into a surface (∼65%) with hydrogen bond donor property and (∼35%) with hydrogen bond acceptor property containing residues like T62, S66, G67, S96, D116, K132, E136, R141, S144, Q145, Q197, K204, N258, S334, S338, S342, T345, and N404. Based on the SiteMap analysis of the two pockets it can be inferred that the substrate binding cavity of the inward-open structure is considerably larger, has more interaction site points, and is highly solvent-exposed as compared to the outward-occluded structure. This observation is consistent with the conformational states of the protein, where in one case the cavity is occluded from the intra and extracellular side, while in the other, it is open and exposed to the solvent from the intracellular side. Nevertheless, despite the discerned differences, both the pockets generated SiteScores >1, where a score greater than 1 suggests a site of particular promise and good druggability^59^ indicating that both the structures could be used for structure-based investigation.

### Molecular docking with glide

**Figure 4:**
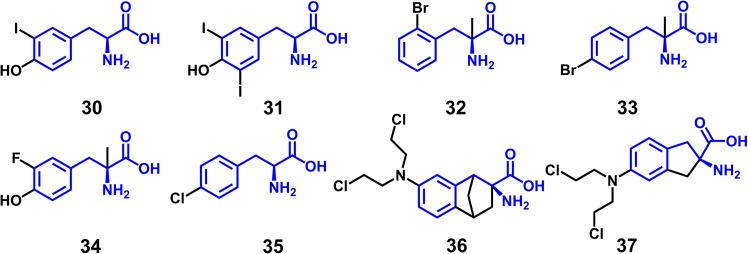
Chemical structures of halogen-containing (X = F, Cl, Br, or I) ligands of LAT1 that were used for the docking studies. **30**, 3-iodotyrosine* **31**, 3,5-diiodotyrosine (pIC_50_: 5.10) **32**, 2-BAMP (2-Bromo α-methyl phenylalanine)* **33**, 4-BAMP (4-Bromo α-methyl phenylalanine)* **34**, FAMT (3-Fluoro α-methyl tyrosine) (pIC_50_: 4.48) **35**, fenclonine* **36**, (±)-2-endo-amino-bis(2-chloroethyl)-7’-amino benzo bicyclo [2.2.1] heptane-2-exo-carboxylic acid (pIC_50_: 5.67), **37**, (±)-2-amino-(bis-2-chloroethyl)-5-aminoindane-2-carboxylic acid) (pIC_50_: 5.30). The common core element ‘2-Amino-3-phenylpropanoic acid’ in **30**–**37** is indicated in blue. *Indicates that the bioactivity data was not reported in term of pIC_50_ values.

As a first step towards identifying the binding mode, we docked a set of recognized halogenated ligands **30**–**37**^20,28,37,60^ of LAT1 (Fig. 4) sharing a maximum common substructure **6** (Fig. 1) into the substrate binding site of the homology model of LAT1 using Glide v6.6^61–63^. The putative binding site was defined using the residues that were identified using SiteMap^58,59^ in the previous section. The substrate translocation in LAT1 is believed to be controlled by a few flexible gating residues, while the other residues surrounding the binding site likely remain rigid and do not undergo significant conformational changes. Hence, we considered the receptor rigid, and the ligands were docked using a softened potential with vdW radii scaling of 0.70 Å in order to reduce the penalty caused by close contacts. The ligands were prepared and minimized using the OPLS-2005 force field within the Schrödinger Suite v.2015-1 prior to docking. To ensure the convergence of conformational sampling, 100 docking poses per ligand were generated with post-docking minimization enabled.

### Evaluation of the docking poses

**Figure 5:**
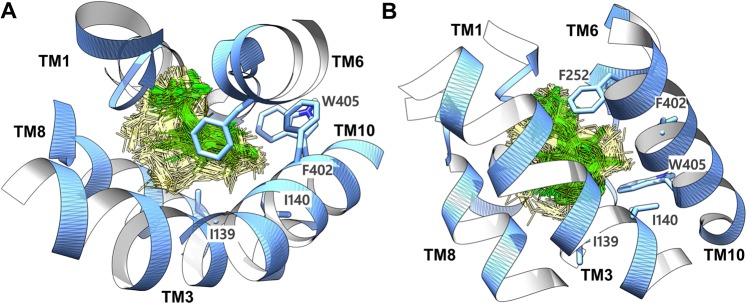
Periplasmic or top-view (**A**) and front view (**B**) of the LAT1 (blue) showing the distribution of 326 CSC’s poses (green) and 474 residual cluster’s poses (yellow). The CSC’s poses are rendered more prominent, while the rest of the residual poses are made less noticeable. Several key residues (F252, I139, I140, F402, and W405) within the binding site are labeled for orientation. The docking poses, and the residues are shown in stick representation, while the protein is depicted in ribbon style.

**Figure 6:**
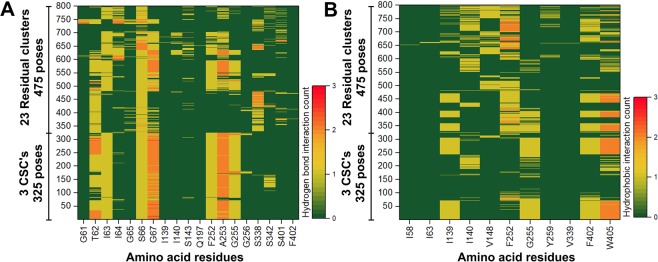
The SIFt representation showing the hydrogen bond count (**A**) and hydrophobic interaction count (**B**) in CSC’s and residual cluster’s poses, excluding electrostatic interactions of the halogen atoms. A continuous pattern of interactions could be observed in the poses belonging to CSC’s as compared to irregular and random interactions in the poses of residual clusters.

For the evaluation of the docking poses, we applied a common scaffold clustering technique which is based on the principle that the ligands that share a common scaffold are believed to exhibit a similar binding orientation^64,65^. The resulting 800 minimized docking poses were clustered according to the RMSD of the heavy atoms of the common substructure **6** (Fig. 1) using an in-house protocol for common scaffold clustering with an RMSD of <2 Å as a similarity threshold^66^. To follow the notion of a common binding orientation, only those clusters were kept that consisted of at least six out of the eight ligands used for docking (common scaffold clusters, CSC’s). This led to the identification of 3 CSC’s and 23 residual clusters corresponding to 326 poses and 474 poses, respectively. Figure 5 shows the distribution of CSC’s and residual cluster’s poses. As can be seen in Fig. 5A,B, the docking poses corresponding to residual clusters (in yellow) are spread over a wide area in the binding site, while the poses belonging to CSC’s (in green) are concerted and oriented in the vicinity of helices 1, 6, and 10. In the next step, we performed Structural Interaction Fingerprint (SIFt) analysis in order to understand the qualitative difference between CSC’s and residual cluster’s poses (Fig. 6). This tool recognizes the residues that display polar or non-polar contacts with the docking poses or are located in close proximity (5 Å) to the ligand. The protein-ligand interaction mapping revealed that the residues positioned on TM1, TM3, TM6, TM8, and TM10 were mostly mediating the binding of the ligands in the docking poses. T62, I63, S66, G67, F252, A253, and G255 were the key residues showing >80% involvement in hydrogen bond interactions in CSC’s poses, while S66 was the sole residue showing >80% interaction rate in residual cluster’s poses. The major residues involved in hydrophobic interactions in CSC’s poses include I139 (41.5%), I140 (17.5%), F252 (11%), G255 (55.6%), F402 (46.1%), and W405 (52.3%). In residual clusters poses I139, I140, V148, F252, G255, F402, and W405 contributed to the binding through hydrophobic interactions and displayed occupancy rates of 28.6%, 30.9%, 30.5%, 68.4%, 18.1%, 40.6%, and 38.1%, respectively.

### The hierarchical clustering approach identified two candidate binding modes

**Figure 7:**
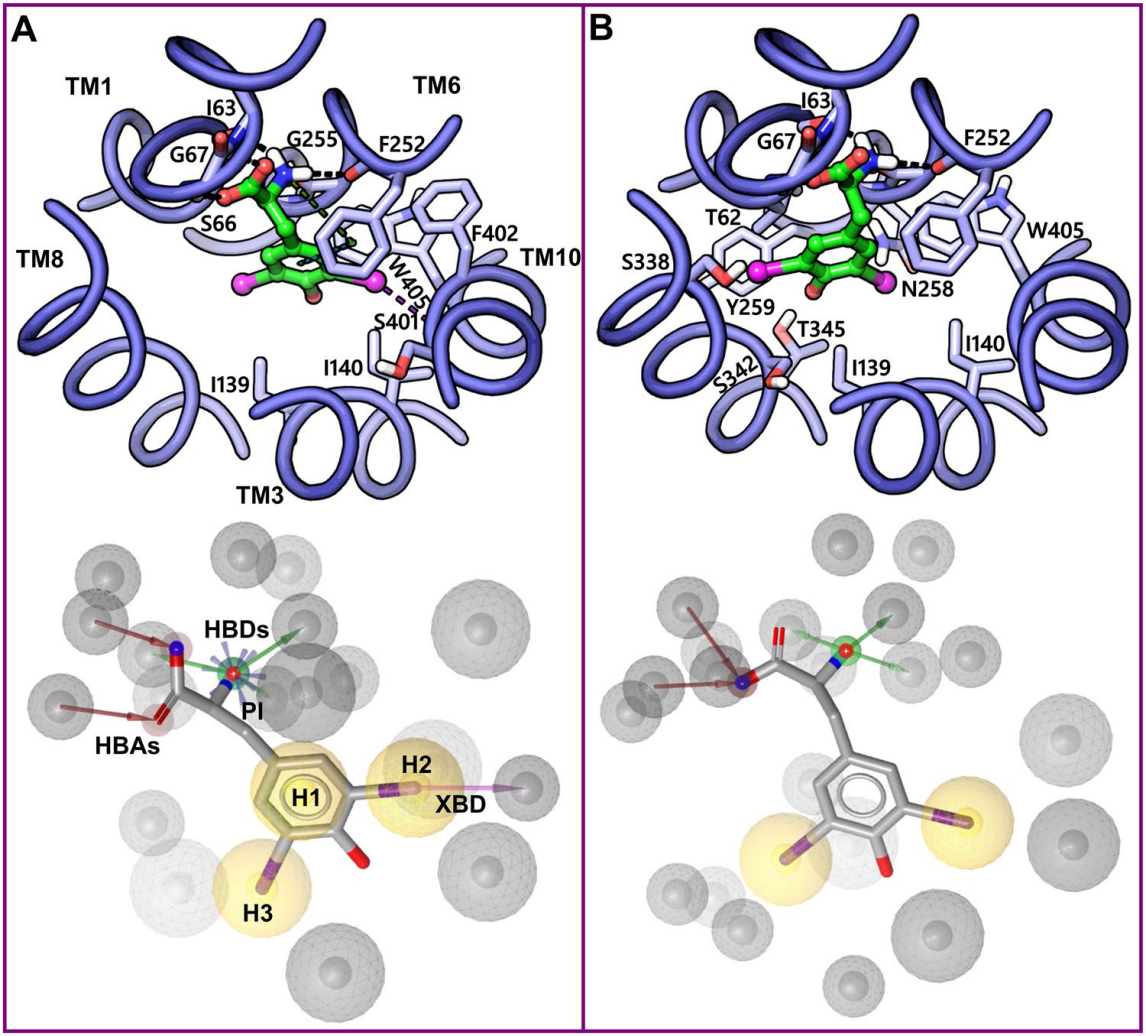
Final clusters of docking poses and the corresponding derivatized pharmacophore models of 3,5-diiodotyrosine **31** in the substrate binding site of LAT1. The five TMs (blue) enclosing the binding site are indicated in the absence of the remaining TMs for the sake of clarity. (**A**) BM I or CSC 1 and 3 poses contain two distinct placements: the amino acid moiety of the ligand is occupying the polar region or ABS of the binding site formed by exposed backbone groups of S66, G67, I63, G255, and F252; and the side chain is placed into the hydrophobic SBS that is enclosed by the residues I139, I140, F252, F402, and W405. (**B**) BM II or CSC 2 poses: amino acid moiety is occupying the ABS; the aromatic side chain is oriented away from the SBS. The protein-ligand contacts were identified using the ligand interactions feature (distance-based) as implemented in Schrödinger. In the docking poses, ligand (green) and residues (blue) are depicted in stick-ball and stick style, respectively. The dotted black, blue, green and purple line in the binding modes indicate hydrogen bond, π-π, cation-π, and halogen bond interactions. In the pharmacophore models (below panel) yellow spheres, green vectors, red vectors, purple vector, and a blue star indicates hydrophobic features (H1-H3), Hydrogen Bond Donors (HBDs), Hydrogen Bond Acceptors (HBAs), Halogen Bond Donor (XBD), and Positive Ionizable group (PI), respectively. The grey spheres are the excluded volume of spheres, and they represent the positions that are sterically occupied by the protein residues. The ligand (grey) is shown in stick style.

**Figure 8:**
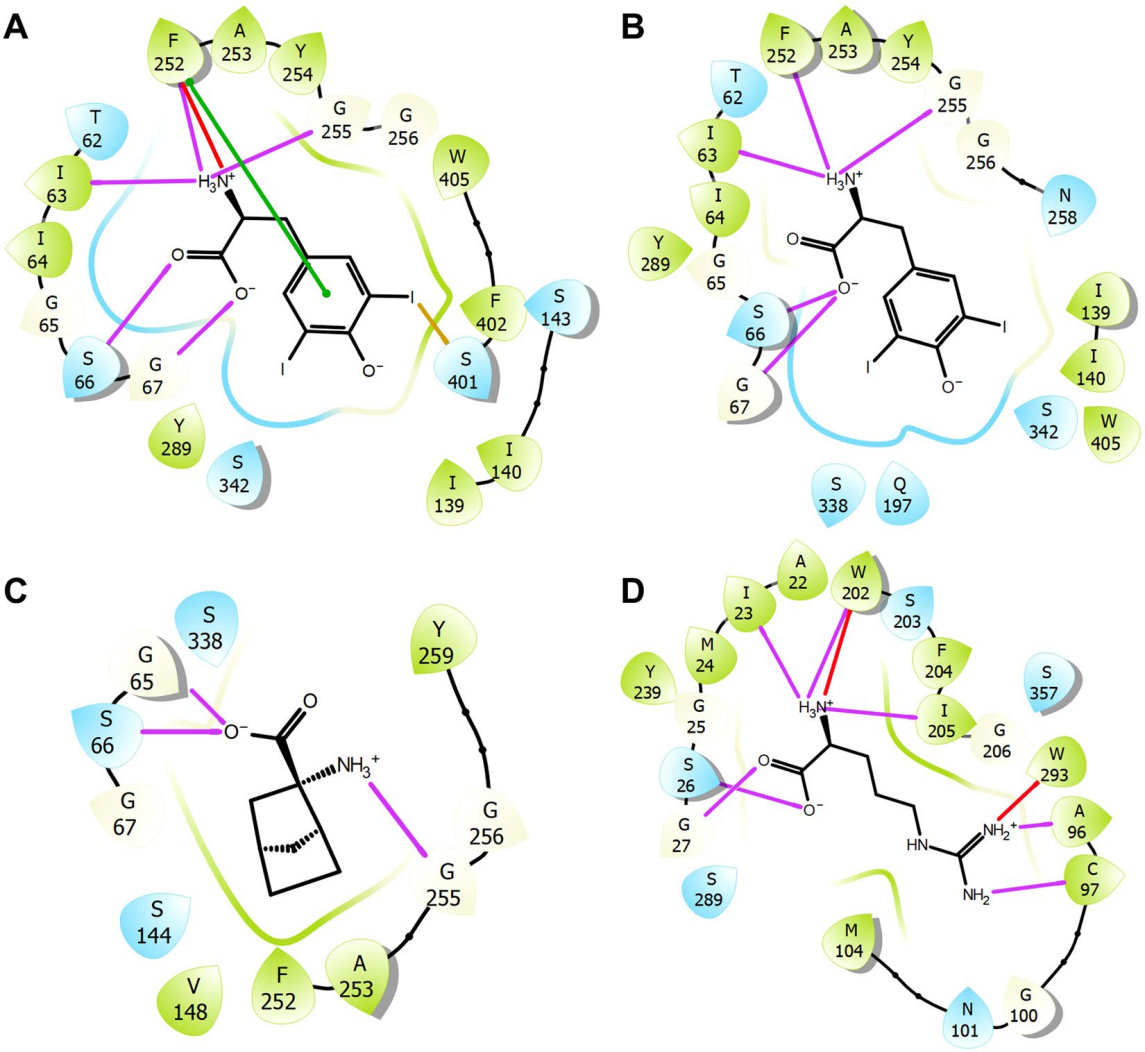
The 2D ligand interaction diagram of BM I (**A**), and BM II (**B**) of **31**; BCH bound to LAT1 in the inward-open state (PDB ID: 6IRT) (**C**); and arginine bound to AdiC (PDB ID: 3L1L) (**D**). The violet, green, red, and yellow lines indicate hydrogen bond, π-π, cation-π and halogen bond interactions, respectively. The polar and hydrophobic residues are highlighted in blue and green, respectively.

**Figure 9:**
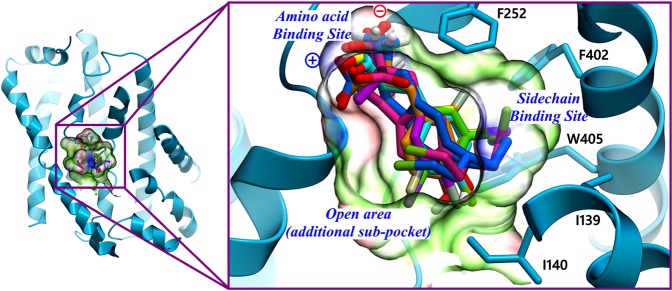
Most favored docking poses of halogenated ligands onto LAT1. Overlay of ligands **30**–**37** as obtained by superposition of the docking poses of CSC 1. The side chain of the ligands occupies the hydrophobic SBS depicted in green, and the amino acid moiety of the ligands is positioned towards the hydrophilic ABS indicated in red and blue. One of the chloroethyl moiety of the nitrogen mustards **36** and **37** is placed near an open and wide region of moderate hydrophobicity and hydrophilicity opposite to SBS that seems to be an additional sub-pocket within the binding site of LAT1. The carbon atoms of the ligand **30**–**37** are colored yellow, green, orange, purple, grey, cyan, pink and blue, respectively.

Highly populated clusters that contained poses of at least six out of eight docked compounds were considered as most promising and thus selected for further analysis. The CSC 1, 2, and 3 included a total of 119, 117, and 90 poses, respectively. Based on the visual inspection of the docking poses of three CSC’s, poses can be grouped into two geometrically different binding modes, BM I corresponding to CSC 1 and 3 (Fig. 7A) and BM II corresponding to CSC 2 (Fig. 7B). For the sake of clarity, it has to be noted that for each cluster, only one representative pose (3,5-diiodotyrosine, **31**) is shown. Interacting amino acids were identified with the function “ligand interactions” from Schrödinger and varied slightly for different poses within one cluster. Docking poses of CSC 1 and 3 (BM I) show five hydrogen bond interactions of the amino acid moiety with the backbone groups of the residues forming the polar region or Amino acid Binding Site (ABS) of the central substrate binding site. The negatively charged α-carboxyl group is accepting two hydrogen bonds from the backbone of S66 and G67 (Figs 7A and 8A) and is positioned in the vicinity of G65 and the side chain of S66 indicating electrostatic interactions. The positively charged α-amino group is donating three hydrogen bonds to I63, F252, and G255 and is stacking against F252 through a cation-π interaction. The phenyl ring is occupying the Side chain Binding Site (SBS) and is involved in an edge-to-face π-π interaction with F252. Also, the meta-substituted iodine (3-I) is donating a halogen bond to the backbone oxygen of S401. Further favorable contacts of the ligand were observed with the residues T62, I139, I140, G255, S342, F402, and W405. In CSC 2 (BM II), the amino acid moiety showed conserved hydrogen bond interactions with respect to BM I though with a slight deviation. The alkoxy oxygen of the α-carboxyl group but not the carbonyl oxygen is engaged in two hydrogen bond interactions involving S66 and G67 (Figs 7B and 8B). The α-amino group is donating three hydrogen bonds to I63, F252, and G255 that are similar to BM I. The RMSD between BM I and BM II is 3.9 Å and the distance between their center of mass is 1.11 Å, indicating that the two BMs differ significantly from each other. The main geometric difference between BM I and BM II that might explain this high RMSD is the orientation of the phenyl ring. In BM I, the phenyl ring is pointing toward SBS and is surrounded by the hydrophobic residues I139 and I140 of TM3, F252 of TM6, F402 and W405 of TM10, while in BM II it is oriented away from the SBS and is partially occupying a wide-open region, opposite to SBS, of moderate hydrophobicity and hydrophilicity surrounded by the residues T62 of TM1, S342, S338, and T345 of TM3, Q197 of TM5, Y259 and N258 of TM6. The loss of hydrophobic interactions of the phenyl ring with F252 in BM II suggests that BM I is more reliable than BM II, since F252 has been reported as a critical gate residue that mediates the binding of the side chain of the substrate^36^. Figure 9 shows the overlay of the docking poses of all ligands corresponding to CSC 1 where the side chain of the ligands was found to occupy the SBS, and the amino acid moieties are placed in the ABS. However, one of the chloroethyl groups of the ligands **36** and **37** is oriented towards the wide region that serves as an additional sub-pocket mediating the binding of the extended and bulky side chain.

Translation and scrutiny of the essential binding features of **31** in terms of a structure-based pharmacophore model further supported our observations from the docking studies. The pharmacophore model derived from BM I exhibited 10 pharmacophoric features: 3 Hydrogen Bond Donors (HBDs), 2 Hydrogen Bond Acceptors (HBAs), 3 Hydrophobic (H1, H2, and H3), 1 Positive Ionizable (PI) group, and 1 Halogen bond donor (XBD) (Fig. 7A, panel below). The 3 HBDs correspond to hydrogen bond interactions of the α-amino group with I63, F252, and G255; 2 HBAs correspond to hydrogen bond interactions of the α-carboxy group with S66 and G67; the PI group signifies the cation-π interaction of the positively charged nitrogen with the side chain of F252. The central hydrophobic feature H1 has F252 and W405 as its interaction partner, while H2 and H3 have I139, F252, F402, W405, T62, and I140 as their interaction partners, respectively. The XBD feature represents the halogen bond interaction of the meta-substituted iodine (3-I) with S401. In the case of BM II, the pharmacophore model showed 7 elements: 2 HBAs, 3 HBDs, and 2 Hydrophobic features (H2 and H3) (Fig. 7B, panel below). The absence of the central hydrophobic feature H1 in this model indicates the loss of aromatic interaction with F252 and W405. The hydrophobic features H2 and H3 correspond to hydrophobic interactions of the meta-substituted iodine (3-I) with I139, I140, and W405 and of 5-I with T62 and I139. Notably, the BM I derived pharmacophore model overlaps perfectly with the previously described pharmacophore model (HBD, Negative Ionizable, Aromatic, and HBA)^47^ and seven features (3 Hydrophobic, 3 HBD and 1 HBA), which is based on a dynamic pharmacophore model of LAT1 ligands, thus emphasizing the trustworthiness of BM I^38^.

**Figure 10:**
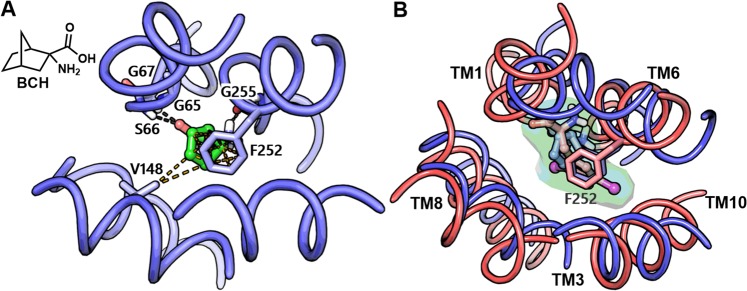
**(A**) binding mode of BCH (green ball-stick) in LAT1 (PDB ID: 6IRT). The black and orange dotted lines indicate hydrogen bond and hydrophobic interactions. (**B**) superposition of the BCH-bound inward-open structure of LAT1 (blue) to the binding mode of **31** (BM I) in the outward-occluded conformation of LAT1 (red). The distance between the center of mass of the ligands (BCH and **31**) is 0.74 Å indicating a substantial volume overlap and high commonality in the binding orientation relative to the neighboring TMs and the interactions of the ligand with the residues in the vicinity of the transporter. The backbone RMSD between the two structures is 3.6 Å, and the alignment score is 0.84.

The recent release of the inhibitor (BCH) bound structure of LAT1 (PDB ID: 6IRT) provided major insights into the binding mechanism. The structure revealed that the amino acid moiety of BCH is involved in three backbone hydrogen bond interactions involving G65, S66 (TM1), and G255 (TM6) (Figs 8C and 10A). The non-polar bicycloheptane ring is engaged in hydrophobic interactions with the side chain of F252 and V148. Importantly, this set of interactions is consistent with the predicted binding mode of **31** underlining the reliability of the docking pose (BM I). The experimentally determined structure of LAT1 reflects the initial ligand recognition state, and it seems that the ligand is not completely bound while the transporter is evolving from the inward-open state to the transient inward-open semi-occluded state. The superposition of the two structures, outward-occluded and inward-open, showed marked conformational changes in TM1, TM6, and TM7, and moderate movement in TM10 resulting in high RMSD of 3.6 Å (Figs 10B and S5). We speculate that the transition of the inward-open state to the occluded state through pivoting of the aforementioned TMs will trigger proper ligand accommodation and stabilize the binding conformation that may reflect our binding hypothesis. In order to assess this supposition, we created a morph using the inward-open structure of LAT1 and the outward-facing occluded model of LAT1. A molecular morph of LAT1 transiting between two conformational states is shown in Movies S1, S2. The key differences in the structures are the conformational changes in TM1/6/7/10 from which TM1, TM6, and TM7 display the most pronounced ones. Additionally, the EL4 of LAT1 swings out during the transition, leading to the expansion of the substrate pathway in the outward-open state. This observation is coherent with the findings of Lee and co-workers, that EL4 might be functioning as an extracellular latch in LAT1 that opens and closes during the transport cycle as observed in other transporters of the amino acid/polyamine/organocation (APC) family^67^. Hence, the rearrangement of EL4 triggers the broadening or narrowing of the substrate pathway leading to antiport flux of substrates. Importantly, structural switching of the transporter from the inward-open to the outward-open or inward-open occluded state, through reorientation of TM1, TM6, and TM7 likely represents a more compact and tight binding state. This can be observed in the morph trajectory, where the inward movements of TM1 and TM6 reduce the volume of the binding site, and the corresponding TMs interact more strongly with BCH via helix backbone interactions. Thus, there is a need for more ligand-bound structures of LAT1 in different conformational states in order to better understand the transport mechanism and binding orientation of the ligands. This would allow benchmarking of docking studies against these structures, which definitely would increase the validity of the binding hypotheses retrieved. Further support in favor of BM I came from the visual scrutiny of BM I with respect to the arginine-AdiC interactions (Figs 8D and S6), which revealed that the majority of the protein-ligand interactions are conserved between LAT1 and AdiC. For example, the amino acid moiety of both ligands is occupying the polar region formed by the exposed backbone groups of the residues located on the helix-break of TM1 and TM6 and is involved in five hydrogen bond interactions. The hydrogen bonding residues I23, S26, G27, W202 and I205 of AdiC corresponds to I63, S66, G67, F252, and G255 of LAT1. The positively charged α-amino group is involved in a cation-π interaction with the side chain of W202 in AdiC and F252 in LAT1. Besides the similarity in ionic interactions, the residues W202 and M104 are mediating the binding of arginine via hydrophobic interactions and they are equivalent to F252 and V148 of LAT1. The stability of the docking pose of **31** (BM I) was also assessed through Molecular Dynamics (MD) simulation in a lipid environment which revealed that the binding orientation was stable throughout the entire simulation (see Supplementary Information, Figs S7–S11). Taken together, the docking poses of the ligands were consistent with the observation of their competitive inhibition of LAT1. Especially, BM I best describes the binding mode of the ligands, where the nonpolar side chain of the ligands points toward the SBS (Fig. 7A), while in BM II, this substructure is oriented away from the SBS (Fig. 7B).

### QM-MM calculations

**Figure 11:**
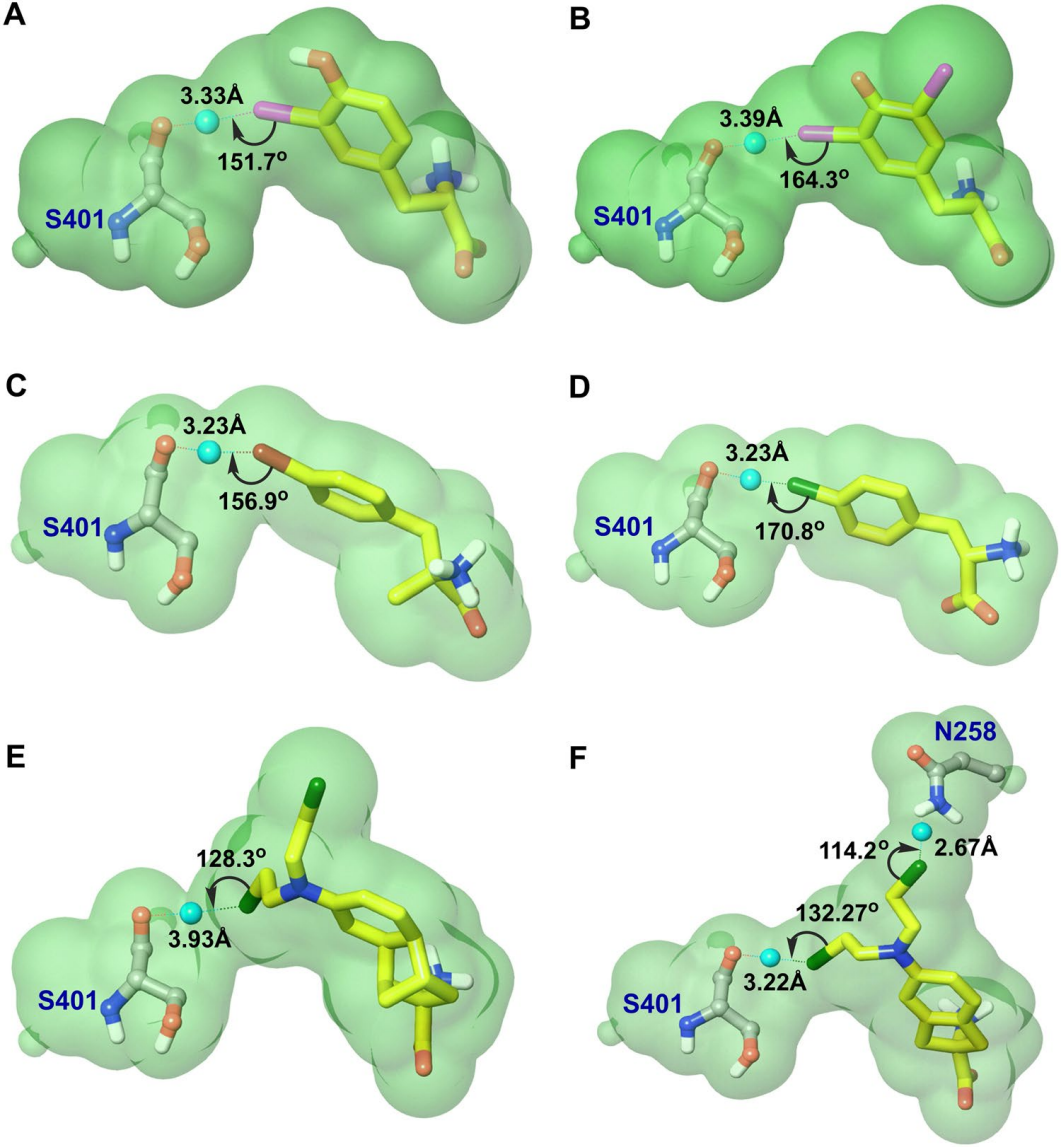
QM-MM optimized poses of **30** (**A**), **31** (**B**), **33** (**C**), **35** (**D**), **36** (**E**), and **37** (**F**) showing electron density (green) mapped on to the surface of the interacting ligand (yellow stick) and residue(s) (grey ball-stick) at the default isovalue of 0.001 electrons/bohr^3^. The bond critical point between the halogen and residue atom is shown as a dummy atom colored in cyan, and the bond path is represented as a zero-order bond to the critical point. The distance between the halogen atom and residue atom, and the σ**-**hole angle are indicated. The ligand (Yellow) and residue(s) (grey) are shown in stick and ball-stick representation, respectively. The rest of the protein is not shown in the optimized poses for the sake of clarity.

The SIFt analysis of the docking poses of **30**–**37** revealed that ∼ 45% of the poses were involved in halogen interactions (Fig. S12). S401 was revealed as a major residue engaged in an X-bond like interaction in 110 out of 124 poses displaying X···O contacts. To explore whether these halogen interactions contribute to affinity improvement, QM-MM optimizations were performed using the docking poses of the ligands. One pose per ligand was selected from the highly populated and common cluster (CSC 1) based on the following criteria as applicable: (i) d(Cl···O)  < 3.27 Å, d(Br···O)  < 3.37 Å, d(I···O)  < 3.50 Å, d(X···H)> 2.8 Å (ii) **∠**C-X···O> 150°, **∠**C=O···X> 90°, **∠**C-X···H> 90°, ∠O-H···X> 120° ^68^ (iii) the best predicted pose in terms of the binding energy that fulfil (i) and (ii). The QM-MM optimization was performed using Qsite^69^ (see Methods), where ligand and residue(s) involved in the interaction were considered at the quantum mechanical (QM) level and the remaining protein at the MM level (Fig. S13). The free energy of binding (ΔG) of the pre- and post-optimized docking poses was calculated using the molecular mechanics-generalized Born surface area (MM-GBSA) algorithm (see Methods). The interaction energy between the ligand and residue was computed via single point energy calculation over the optimized QM core. The results from the QM-MM and interaction energy calculations are summarized in Tables S1 and S2. Figure 11 shows the optimized poses, where X-bond or hydrogen bond interactions are observed by the presence of a bond critical point between halogen and residue atom. The critical point between the two atoms indicates that the interaction is favorable^70^. The QM-MM optimization resulted in a more stable geometrical configuration of the X-bond interaction in **30**, **31**, **33**, and **35**. However, an unfavorable change in the geometry of the X-bond was observed in **36**, where the distance d(Cl···O) increased from 2.91 Å to 3.93 Å and the σ-hole angle **∠**C-Cl···O remarkably decreased from 162.4° to 128.3° after the optimization. A similar change in donor and acceptor angle was observed in **37** indicating a weak X-bond interaction. The RMSD of ligands after the optimization was <1 Å, indicating that the predicted docking pose was conserved. The geometry optimization significantly improved the binding energy of the poses and is denoted by ΔΔG (ΔG_QM-MM_ − ΔG_Glide_) (Table S1). The Spearman rank coefficient **r**_**s**_ between ΔG_Glide_ and ΔG_QM-MM_ was 0.90, indicating a good correlation between the binding energies. The **r**_**s**_ between E_QM-MM_ and ΔG_Glide_ was 0.67, and **r**_**s**_ between E_QM-MM_ and ΔG_QM-MM_ was 0.52, indicating a moderate correlation (Fig. S14). The regression coefficient **r**^2^ between ΔG_QM-MM_ and pIC_50_ of **31**, **34**, **36**, and **37** was 0.72. To account for the bias of selecting a best-predicted pose for the optimization, we selected a random pose of **31**, without geometrical constraints, showing interaction with S401 for the QM-MM. After optimization, we observed the loss of X-bond interaction as revealed by the absence of a bond critical point (Fig. S15). Additionally, the ΔG_QM-MM_ of the random pose was less than the best-predicted pose of **31**, indicating that the X-bond interaction is contributing to the binding energy. To further elucidate the X-bond interaction, which is impelled by the electrostatic attraction between the electropositive σ-hole of halogens and the electronegative atom, we calculated the maximum electrostatic potential (V_S,max_) of the halogen atoms (see Methods) (Fig. S16, Table S2). As expected V_S,max_ decreased in the order I > Br > Cl. In **31**, the presence of the deactivating iodine 5-I on the aromatic ring resulted in a large positive potential on 3-I (V_S,max_ = 42.79 kcal mol^−1^) as compared to **30** (V_S,max_ = 38.44 kcal mol^−1^). The para substituted bromine in **33** showed intermediate potential (V_S,max_ = 20.73 kcal mol^−1^). The para substituted chlorine in **35** showed higher potential (V_S,max_ = 11.93 kcal mol^−1^) than the aliphatic chlorines in **36** and **37** (V_S,max_ = 4.37 and 4.79 kcal mol^−1^, respectively). The interaction energy (ΔE) between the ligand and S401 decreased in the following order: **31** (ΔE = −4.96 kcal mol^−1^) > **30** (ΔE = −2.69 kcal mol^−1^) > **33** (ΔE = −2.33 kcal mol^−1^) > **35** (ΔE = −1.85 kcal mol^−1^) > **37** (ΔE = −0.99 kcal mol^−1^) > **36** (ΔE = −0.85 kcal mol^−1^). The low ΔE of **36** and **37** could be attributed to the improper X-bond geometry and poor V_S,max_ on chlorines making the interaction unfavorable. The ΔE of **32** with S143 was −3.32 kcal mol^−1^ (Fig. S17), and the ΔE of **37** with N258 was −1.44 kcal mol^−1^. Interestingly, Yan and coworkers recently demonstrated that N258A mutation leads to a marked decrease in transport activity of LAT1, indicating the importance of this residue in substrate binding and translocation^32^. Furthermore, N258 is believed to be a distal gate element of LAT1 that corresponds to E208 of AdiC engaged in the inter-residue hydrogen bond network^57^. The correlation of ΔE with V_S,max_ has been shown numerous times^45,71,72^, and in this investigation, we obtained an **r**^2^ of 0.83 (Fig. S14) indicating the reliability of results obtained from the QM-MM optimization. Overall, the docking simulations showed that the ortho position is the least favorable for halogen interactions compared to the meta (highly favorable) or para (intermediate) positions as revealed by frequency analysis of the halogen contacts for different ligands (Fig. S12). The QM calculations revealed that the more polarized aromatic halogens show strong X-bond interaction, while the less polarized aliphatic halogens show weak X-bond interaction. Recently, Augustyn *et al*. have shown that meta-substituted halogens display an inhibition trend I > Br > Cl> F in phenylalanine and Br > Cl = F > I in tyrosine^48^. It seems likely that the halogens in these analogs and others (**1**–**5**) exhibit polar interactions (X···O or X···H) with the binding site residues of LAT1. Also, these halogenated ligands could be made more affine by adding electron-withdrawing substituents, such as fluorine, that would intensify the positive potential of the σ-hole, and consequently the strength of the halogen interactions.

**Figure 12:**
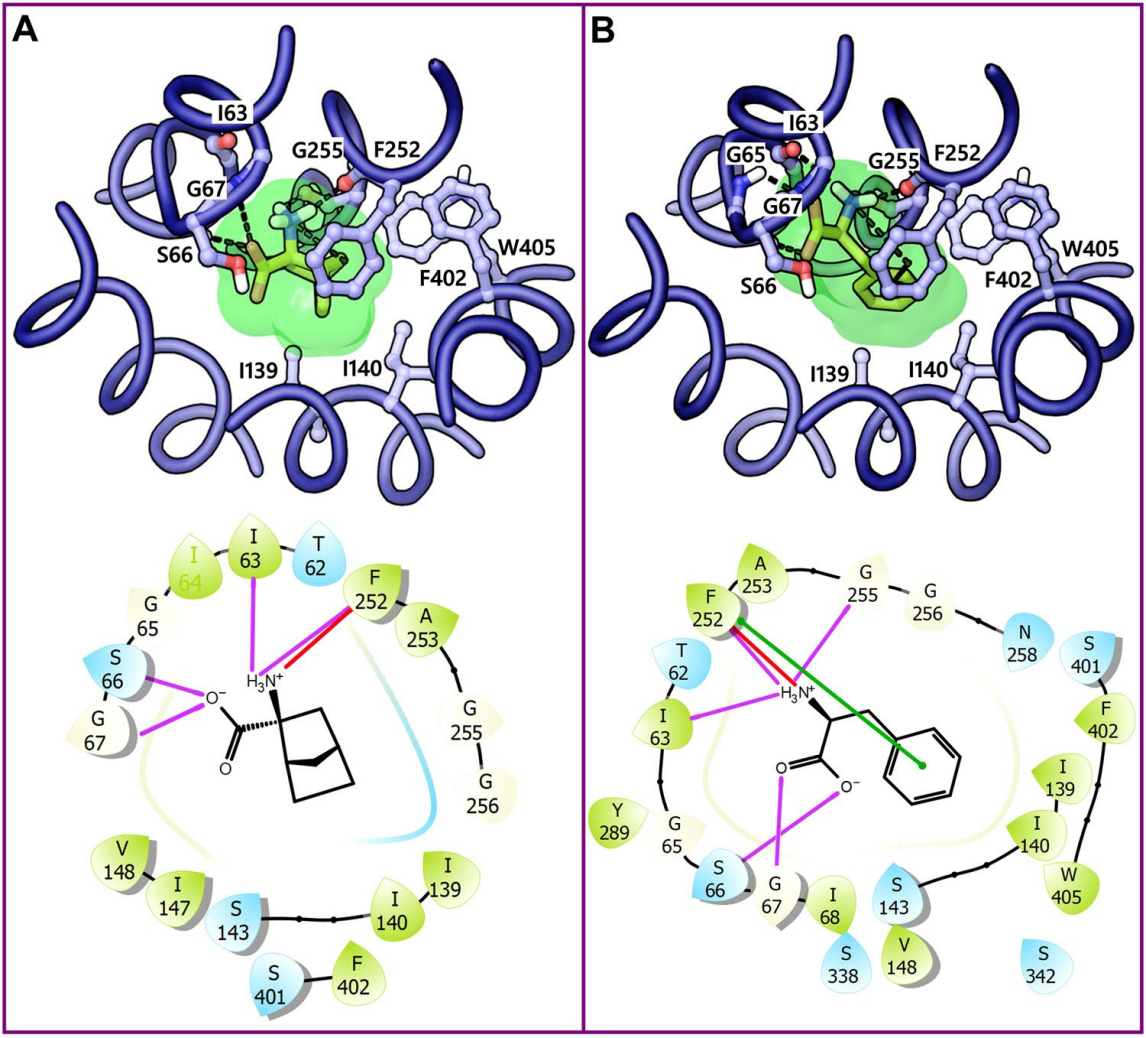
QM-MM optimized poses and the corresponding 2D ligand interaction diagram of BCH (**A**) and L-phenylalanine **6** (**B**) to LAT1 (blue ribbon); the electron density (green) is mapped on to the surface of the ligand at the default isovalue of 0.001 electrons/bohr^3^. The ligand (yellow) and the residues (blue) in the docking poses are shown in stick and ball-stick representation, respectively. The protein-ligand contacts in 2D interaction maps are highlighted according to Fig. 8.

We also performed the docking and Density Functional Theory (DFT) calculations of BCH and L-phenylalanine **6** in order to compare the results obtained using halogenated ligands. Figs 12A,B show the optimized pose of BCH and **6** to the outward-occluded model of LAT1. The binding geometries of the ligands are similar to BM I as revealed by preserved ionic interactions of the amino acid moiety and hydrophobic interactions of the side chain. The optimized binding energy of BCH and **6** is lower than that of the halogenated analogs, except the fluorine derivative FAMT (**34**; Table S1). FAMT did not exhibit halogen interactions in the docking poses, which may be attributed to the lack of positive areas on the surface owing to the high electronegativity of the fluorine atom. This observation is in agreement with the decreased activity of **34** compared to other ligands. The visual inspection of the two binding orientations of BCH (outward-occluded and inward-open) revealed that BCH in the outward-occluded state is packed more tightly within the proximal pocket, enclosed by the residues G255 and F252, compared to the inward-open structure with an RMSD of 3.2 Å. The key difference between the two binding modes is related to the interaction of the amino acid moiety. In the outward-occluded structure, this moiety is placed favorably to engage in five polar contacts (Fig. 12A) compared to three contacts (Figs 10A and 8C) in the inward-open state. Thus, we presume that the structural shift from the inward-open to the occluded sate through movements of the TMs of the core domain (TM1/2/6/7) may stimulate favorable adaptation of the ligand leading to stronger binding. Altogether, the QM-MM study provides strong evidence for an X-bond or a hydrogen bond interaction of the halogens that are promoting the ligand binding, and these interactions may be contributing to the inhibitor selectivity (particularly meta-substituted halogens that exhibited better halogen bond geometry).

## Discussion

LAT1 plays a vital role in cancer progression, and inhibiting its transport function is therapeutically beneficial. In this study, we constructed a homology model of LAT1 based on the crystal structure of AdiC (PDB ID: 3L1L) in the outward-occluded conformation. Remarkably, the constructed model is in excellent agreement with the recently released cryo-EM structures (PDB IDs: 6IRT, 6JMQ) of LAT1^12,32^ in the inward-open state which revealed that its substrate binding site consists of an ABS that is characterized by a positive pole in TM1 and a negative pole in TM6 that mediates the binding of the complementary negatively charged carboxyl group and the positively charged amino group of the ligand (Fig. 9). The positive and the negative pole are generated by the exposed backbone groups of residues G65, S66, G67, F252, A253, and G255. In addition, it has an SBS that mediates the binding of the side chain of the ligand. The SBS is a large and wide pocket that can accommodate both the primary side chain as well as bulky substituents on the meta or flexible substituents on the para position of the aromatic ring. F252 and G255 form the proximal part of this pocket, while the distal portion of this pocket is comprised of several hydrophobic residues such as L251, W257, W405, and V408^12^. The presence of a smaller residue G255 in LAT1 allows the meta substituents to access the distal pocket which is believed to be obstructed in AdiC due to steric barrier induced by the bulky side chain of equivalent residue I205^12^. The promiscuous nature of LAT1 pocket has been lately demonstrated, where polar substitutions on the meta position of phenylalanine displayed a significant affinity for LAT1^50^. The presence of a polar residue N404 in SBS may account for possible hydrogen bond interactions with the meta-substituted polar moiety and thus explains the promiscuity of LAT1. The binding site comparison of the inward-open and the outward-occluded structures revealed that the two pockets are distinct in terms of volume and shape. The pocket corresponding to the outward-occluded structure is small and less exposed to the solvent, whereas the pocket of the inward-open structure is large and more solvent-exposed. The reduced exposure of the outward-occluded pocket indicates that the bound ligand is more buried compared to the inward-open structure. Nevertheless, both the structures were found equally promising for carrying out structure-based studies.

Our common sub-structure guided docking protocol and pose estimation of halogenated ligands led to the identification of two candidate binding poses, BM I and BM II. The poses corresponding to BM I were positioned close to the TM1/6/10 interface and this observation was reflected in the SIFt analysis where residues I63, S66, G67 (TM1), F252, G255 (TM6), F402 and W405 (TM10) showed significant occupancy rate. While the poses pertaining to BM II were distributed over a broad region in the substrate binding site exhibiting varying interaction pattern with the residues of TM1/3/6/8/10. With respect to the interaction profiles of BM I and BM II, we found similar interactions of their amino acid moiety with the main-chain atoms of TM1 (S66, G67, I63) and TM6 (F252, and G255). Consistent with our binding hypothesis, Lee at al. have shown that G65A, S66V, S66N, and G67A mutations sharply reduces the leucine uptake in LAT1 expressing *Xenopus laevis* oocytes indicating their importance in substrate recognition through backbone interactions^12^. In BM I, the aliphatic portion of the side chain (α and β) and the phenyl ring show strong hydrophobic interactions with F252 and G255, while in BM II the side chain of the ligand is positioned away from F252 resulting in reduced hydrophobic interactions. The importance of F252 for the substrate binding has been shown previously where F252A mutation completely abolished the transport activity of LAT1 indicating that F252 is likely functioning as a proximal gate element allowing substrate entry in the binding site^12,32,36^. F252 of LAT1 corresponds to F231 in ApcT^73^, F253 in LeuT^74^ and W202 in AdiC^75^, all of which have been shown to play a critical role in the substrate binding. Hence the hydrophobic interaction involving F252 seems to be an energetically significant interaction mediating the binding of the side chain. These observations might indicate that indeed BM I is more promising than BM II to account for the binding of ligands to LAT1. Docking pose of **31** also revealed that the meta-substituted iodine is donating a halogen bond to the backbone oxygen S401. Importantly, many halogen interacting residues such as E136, F252, S338, and S401 are within the binding site of the LAT1 structure that might strengthen our hypothesis of non-covalent interactions of the halogens playing an important role in the ligand binding. Further post-hoc analyses of BM I with respect to the BCH bound structure of LAT1 revealed a tight ligand volume overlap upon superposition and a common residue interaction profile of the ligands; the amino acid moiety is engaged in hydrogen bond interactions with the residues located in the helix break of TM1 and TM6, and the side chain (bicycloheptane ring in BCH and phenyl ring in **31** is interacting with F252. Hence, the structure of LAT1 strengthens the BM I-like binding orientation of the ligands in LAT1. Also, the predicted model of LAT1 and the docking pose of **31** (BM I) was found to be stable during the MD simulation (see Supplementary Information, Fig. S7–S11). The pharmacophore modeling suggests that a common pharmacophore -the minimum set of structural and functional features required for the effective ligand binding to LAT1- should contain five hydrogen bonding features and a central hydrophobic feature. The presence of additional planar or nonplanar hydrophobic feature corresponding to the meta position of the aromatic ring is beneficial for affinity. Notably, the meta-substituted halogen (I, Br, or Cl) plays a dual role by serving as both hydrophobic and halogen bonding feature. In total, our docking protocol led to the identification of one convincing binding mode (BM I) that may account for the binding of amino-acid related ligands in LAT1.

In order to investigate whether halogen interactions contribute to affinity, we selected the docking poses of **30**–**37** that meet the optimum geometry requirements of a non-covalent interaction with the halogen. This was followed by optimization of the poses with DFT using hybrid QM-MM, and subsequently interaction energy calculations over the optimized QM core. The combination of molecular docking and QM-MM methods can make a potentially important contribution by allowing modeling and estimation of specific non-covalent effects of the ligands such as halogen or metal interactions. The QM calculations indicated a stable and attractive X-bond interaction in **30**, **31**, **33**, and **35**, contributing to affinity. The X-bond interaction energy trend **31 > 30** > **35** is consistent with the *in vitro* inhibition order **31 > 30** > **35**^37^. Several experimental structures have shown that halogens play an essential role in ligand recognition. For instance, the binding mode of T_4_ in its transport protein transthyretin (PDB ID: 1IE4) revealed that the iodine atom of T_4_ is engaged in two short polar interactions with the backbone of transthyretin (Fig. S18A)^76^. In another case, the crystal structure of glutamate receptor 2 (PDB ID: 1MY4)^77^ bound to 5-iodowillardiine, a heterocyclic amino acid, demonstrated that the iodine is involved in an X-bond interaction with the sulfur of M196 and a hydrogen bond interaction with the side chain of T174 (Fig. S18B). Furthermore, X-bonding has been successively exploited for designing anticancer drugs targeting the p53 pathway^78–80^ and inhibitors of PDE5^81^. Recently, in an elegant study, Shinada *et al*. through meticulous analysis of a large dataset of protein-ligand complexes showed that I, Br, and Cl exhibited significant frequency of polar contacts with the protein residues highlighting the importance of halogen interactions in the binding process of small molecules^82^. Since T_4_, T_3_, reverse T_3_, and 3,3’-diiodothyronine (3,3’-T_2_) are known LAT1 ligands^27,39^, it is likely that they show the X-bond interaction of polarized iodine(s) that may explain their high affinity. These observations suggest that the halogens could be exploited as a chemical tool to boost affinity and selectivity in the rational design and synthesis of new inhibitors of LAT1.

In conclusion, we showcased here a structure-based strategy that incorporates an exhaustive sampling of docking poses and their valuation with respect to the common scaffold to elucidate the binding hypothesis of LAT1 ligands. In particular, we put an emphasis on understanding the non-covalent interactions of the halogen atoms by employing QM-MM, which we think might be playing an essential role in augmenting the ligand binding and conferring selectivity. Despite the good overlap of docking results with the inward-open structure of LAT1, we observed an important difference with respect to the residue interaction of amino acid moiety of the ligands. In the inward-open structure (PDB ID: 6IRT), the amino acid moiety of BCH is involved in three hydrogen bond interactions (Figs 8C and 10A), while in the outward-occluded model this moiety is oriented favorably to engage in five (**31**, Figs 7A and 8A) or four (BCH, Fig. 12A) hydrogen bond interactions and a cation-π interaction possibly signifying a high-affinity binding. Therefore, docking studies against the inward-open structure may be useful to comprehend the ligand-binding profiles in detail. This might lead to a better understanding of the orientations and the interactions governing ligand recognition in different states of the transport cycle. Overall, the findings attained here may be useful for designing critical experiments that might help to establish the role of residues in substrate binding (for example, E136, S401, and W405). The flexible side chain docking could be considered as a methodological advancement to the contemporary approach that may be valuable to identify the crucial residues involved in the binding and gating mechanism in LAT1. Finally, the knowledge gained from this study combined with the availability of the cryo-EM structures of LAT1 will reinvigorate the detailed investigations of the binding modes of inhibitors and the discovery of novel small molecule inhibitors through structure- and experimental-based approaches.

## Methods

### Homology modeling

The high-resolution X-ray structure of the arginine-agmatine antiporter AdiC in the outward-occluded conformation (PDB ID: 3L1L) served as the template for building a protein homology model. The sequence alignment between AdiC and human LAT1 (UniProt accession number: Q01650) was adapted from our last study^38^. One hundred homology models were constructed using MODELLER 9.14^83^. The top-scored model with respect to DOPE score was selected for the docking studies^55^. We also verified that the model was reliable with the results from previous mutagenesis studies, in particular with respect to the location and orientation of residues important for ligand interaction such as F252 and S342^36^. The model was subjected to automated structure preparation using the Protein Preparation Wizard in the Schrödinger Suite in order to optimize the hydrogen bonding network, and enable the protonation of titratable residues and permitting the flipping side chains of Asn, Gln, His^84^. Finally, the structure was energy minimized by keeping the backbone constrained using the OPLS-2005 force field^84^. The stereochemical quality of homology modeling was evaluated by the Ramachandran’s plot assessment using PROCHECK^85,86^.

### Morph of conformational changes in LAT1

Conformational changes involved in the transition of LAT1 from the inward-open to the outward-occluded state were morphed using the solved structure of LAT1 (PDB ID: 6IRT) and the AdiC (PDB ID: 3L1L) based outward-occluded model of LAT1. The two structures were superpositioned, and the morph of the structures was created using the University of California, San Francisco Chimera package (www.cgl.ucsf.edu/chimera/).

### Docking

The ligand preparations were performed using LigPrep^87^ in the Schrödinger Suite v.2015-1. Protonation states were calculated at target pH 7.0 ± 2.0 and thirty-two stereoisomers were computed for each ligand by retaining specified chiralities. Docking is based on a grid characterized by physical properties in the receptor volume that is explored for ligand-receptor interactions during the docking procedure. The “Receptor Grid Generation” panel of Glide was used to prepare the Grid files with Grid points calculated within a region or an enclosing box defined with a set of predicted binding site residues. Docking was performed using the Glide module of Schrodinger. The scoring function of Glide named ‘Glidescore’ was used for the binding affinity prediction. The docking/scoring can be performed using either standard (SP) or extra precision (XP). The enhancement of XP over SP includes the addition of large desolvation penalties to both ligand and protein, assignment of specific structural motifs that contribute significantly to binding affinity, and expanded sampling algorithms needed by scoring function enhancement. The XP scoring function includes four elements: E_coul_ (Coulomb energy), E_vdW_ (van de Waals energy), E_bind_ (components favoring binding), and E_penalty_ (components impeding binding). In this study, XP docking was applied, and 100 conformations (or poses) per ligand were generated. LigandScout 4.4 (Inte:Ligand GmbH) was employed to generate the pharmacophore models from the docking poses; default settings were used for pharmacophore modeling^88^.

### Common scaffold clustering

The docking poses were hierarchically clustered according to the heavy atoms of **6** using the cheminformatics utility ‘Clustering of Conformers’ of Maestro^89^. The clustering was performed by applying the complete linkage algorithm with a clustering height of 2.0 Å. Only those clusters were considered that contained poses of at least six out of the eight ligands docked. The poses corresponding to common scaffold clusters were analyzed and visualized in Maestro^89^.

### Structural Interaction Fingerprint (SIFt) analysis

The “Interaction Fingerprints Panel” of Maestro was used for deriving the Interaction fingerprints (IFPs) for the docking poses as described previously^90,91^. This method describes the presence or absence of noncovalent interactions (hydrogen bond and hydrophobic interactions) between the ligand and the binding site residues by using bits. In this study, a distance cutoff of 5 Å between heavy atoms was defined for the binding site, and the interacting set comprises the residues that contain atoms within the specified cutoff distance from the ligand atoms. An interaction matrix is then constructed, including the bits with appropriate information of the defined chemical interactions.

### MM-GBSA calculations

The Molecular Mechanics-Generalized Born Surface Area (MM-GBSA) approach represents the postprocessing method to evaluate free energies of binding and for carrying out geometry minimization that combines molecular mechanical energies with continuum solvent approaches^92,93^. The binding energy (ΔG_bind_) can be expressed by Eq. 1, where G_complex_, G_protein_, and G_ligand_ signifies the free energy of the complex, energy of the protein without the ligand and energy of the unbound ligand, respectively.

1 $${\Delta G}_{\mathrm{bind}}={{\rm{G}}}_{{\rm{complex}}}-{{\rm{G}}}_{{\rm{protein}}}-{{\rm{G}}}_{{\rm{ligand}}}$$

The free energy of the complex, protein, or ligand is a sum of nonbonded electrostatic interactions, van der Waals, internal strain, and solvation energy terms. These parameters were calculated by using the VSGB2.0 implicit solvation model and OPLS-2005 in Prime^7^. The entropic term associated with the protein or ligand is not considered by default. However, the solvent entropy term is implemented in the VSGB2.0^93^.

### QM-MM calculations

Docking poses were optimized using QM-MM, where QM core consisted of ligand and residue(s) interacting with a halogen atom and the rest of the protein was considered in the MM region. Cuts between the QM and MM region were treated with the frozen-orbital method as implemented in Qsite^94,95^. All QM calculations were performed using restricted DFT method with the B3LYP functional and LACVP* basis set. Convergence is based on energy change (5.00 × 10^−5^ Hartree), and root mean square density matrix change (5.00 × 10^−6^). The MM region was treated with OPLS-2005, and the energy was minimized using the Truncated Newton Conjugate Gradient (TNCG) method. Convergence criterion is based on energy change (1.0 × 10^−7^ kcal/mol) and gradient (0.01 kcal/mol Å). The nonbonded interactions (electrostatic and van der Waals) cutoff is set to 10 Å while the dielectric constant *ϵ* in the gas phase is set to unity. The maximum step size and cycles for iteration during minimization were 1.000, and 1000, respectively. The QM-MM optimization was carried out without any constraints in vacuum. Single-point energy calculations were performed separately to determine the interaction energy (ΔE) between the ligand and residue in the QM layer using the same level of theory as applied for the geometry optimization. All calculations were carried out using Jaguar 8.7^96^.

### Electrostatic potential on a molecular surface

The electrostatic potential (ESP) V(r) around a molecule can be calculated meticulously by using Eq. 2 where Z_A_ is the charge on nucleus A, located at R_A_, and ρ(r) is the molecule’s electron density. V(r) is a physical observable which can be determined both experimentally using diffraction techniques, and computationally.

2 $${\rm{V}}({\rm{r}})=\sum _{{\rm{A}}}\frac{{{\rm{Z}}}_{{\rm{A}}}}{|{{\rm{R}}}_{{\rm{A}}}-{\rm{r}}|}-\int \frac{{\rm{\rho }}({\rm{r}}^{\prime} )\,d{\rm{r}}^{\prime} }{|{\rm{r}}^{\prime} -{\rm{r}}|}$$

Its sign in any region of space depends upon whether the positive contribution of the nuclei or the negative contribution of the electrons is dominant. When plotted on the surface of a molecule (electron density ρ = 0.001 au)^68,97^ V(r) is designated V_S_(r), and its local most positive and most negative values are identified as V_S,max,_ and V_S,min_. In this study, V_S_(r), V_S,max_ and V_S,min_ were calculated using Spartan 14^98^.

### Molecular dynamics

Molecular dynamics (MD) simulation (20 ns) was performed in GROMACS v.5.0.5^99^ using united atom GROMOS 54a7 force field. The topology and coordinates of dipalmitoylphosphatidylcholine (DPPC) were taken as described by Peter Tieleman (http://wcm.ucalgary.ca/tieleman/downloads)^100^. The receptor-ligand complex was placed at the desired position and orientated to the OPM^101^ generated receptor overlapped to the lipid bilayer DPPC using PyMOL^102^. The coordinates of the complex and DPPC was saved, and lipids were packed around an embedded protein using the INFLATEGRO script^103^. Overlapping lipids were deleted, and alternate compression and energy minimization were performed to achieve the desired results. The compressed system was hydrated using simple point charge water model^104^ with periodic boundary conditions. The Genion utility of GROMACS was used to add 8 Na^+^ and 10 Cl^−^ to the system to satisfy the electroneutrality condition. The production simulation was performed at a constant temperature, pressure, and a number of particles (NPT). Long-range electrostatic interactions were estimated by using the particle mesh Ewald method with a 0.16 nm cutoff for the real space calculation^105^. A cutoff of 1.2 nm was used for the van der Waals interactions. The Nosé-Hoover thermostat^106,107^ was applied at 323 K with coupling constant τ = 0.5 ps. The velocities and coordinates were saved every 2 ps. The timestep for integration was 2 fs, and the LINCS algorithm was used to restrain bond lengths^108^. RMSD and RMSF relative to the initial structure were calculated using the gmx rms and gmx rmsf tools of GROMACS.

## Supplementary information

Supplementary Information

Movie S1

Movie S2

## Data Availability

The data supporting the findings of this study can be found in Supplementary Information files and from the corresponding author upon request.
