# Supplementary material for: Rigorous sampling of docking poses unveils binding hypothesis for the halogenated ligands of L-type Amino acid Transporter 1 (LAT1)
Source: Sci Rep. 2019 Oct 21;9:15061. doi: 10.1038/s41598-019-51455-8 (PMC6803698; doi:10.1038/s41598-019-51455-8)
Supplement: Supplementary file 1 — Supplementary Information [file 41598_2019_51455_MOESM1_ESM.pdf]

Supplementary Information

Rigorous sampling of docking poses unveils binding hypothesis for the halogenated ligands of L-type Amino acid Transporter 1 (LAT1)

Authors: Natesh Singh,[1] Bruno O. Villoutreix,[1] Gerhard F. Ecker[2,*]

[1]University of Lille, Inserm, Institut Pasteur de Lille, U1177 - Drugs and Molecules for living Systems, F-59000 Lille, France

[2]Department of Pharmaceutical Chemistry, University of Vienna, Althanstrasse 14, 1090 Wien, Austria

Corresponding author: gerhard.f.ecker@univie.ac.at

Contents

Hydrophobicity and Electrostatic potential analysis 3
Molecular dynamics 3-4
Figure S1 4
Figure S2-S3 5
Figure S4 6
Figure S5 7
Figure S6 8
Figure S7-S8 9
Figure S9-10 10
Figure S11 11
Figure S12 12
Figure S13 13
Table S1-S2 14
Figure S14 15
Figure S15-S16 16
Figure S17-S18 17
References 18

Hydrophobicity and Electrostatic potential analysis

The hydrophobicity of the LAT1 model was compared with the AdiC template by relying on the Kyte-Doolittle hydrophobicity scale.[1] The hydropathy profile was similar in both structures that further validates the LAT1 model (**Figure S1**, **S2A**-**B**). The adaptive Poisson Boltzmann Solver version 1.3 (APBS)[2] was used for generating the electrostatic potential surface (EPS) of LAT1 and AdiC. The PQR files were created from the PDB coordinates using PDB2PQ[3,4] (V. 2.0) and AMBER forcefield.[5] PROPKA[6] was utilized to determine the protonation state and radius of the distinct atoms at pH 7.0. The pH-specific PQR file was then used to compute the electrostatic surface charge distribution with a Linearized Poisson-Boltzmann (PB) equation and cubic B-spline discretization of the charge distributions. PB calculations were performed at 298 K with a dielectric constant of 78.0 for water and 4.0 for the protein interior. The ion concentrations were set to 0.015 M with an ionic radius of 2.0 Å. Ion accessibility was defined using inflated van der Waals radii. The dielectric coefficient was determined using the molecular surface definition with simple harmonic average smoothing[2]. The EPS of the transmembrane domain of LAT1 was mainly neutral with some positive and negative regions (**Figure S2C**), while the EPS of AdiC was generally positive with some neutral areas (**Figure S2D**).

Molecular dynamics

The conformational stability of the predicted structure of LAT1 and of the docking pose of the 3,5-diiodotyrosine **31** was assessed by analyzing the trajectories obtained in 20 ns simulations. The MD simulation of the 3,5-diiodotyrosine-LAT1 complex showed that after an initial rapid jump Cα Root Mean Square Deviation (RMSD) plateaued at 0.34 nm after ~ 2 ns, with only subsequent small fluctuations thereafter and a final value after 20 ns of 0.33 nm (**Figure S7A**). The average pairwise Cα RMSD was 0.22 ± 0.05 nm with no significant conformational change observed in the ensemble members indicating a similar α-helices packing (**Figure S8**). To determine protein regions showing high flexibility, the Root Mean Square Fluctuation (RMSF) per residue was calculated for the last 10 ns. Higher local fluctuations occurred in the loop regions, particularly in residues 364-372 connecting TM8 and TM9. The largest flexibility was observed in loop residues 389-393 linking TM9 and TM10 (**Figure S7C**). Other residues showing fluctuations were mainly located in the intra and extracellular loops. Minimum RMSF was observed in the protein core and residues of the binding site. The protein secondary structure

analyzed by using DSSP (Define Secondary Structure of Proteins)[7,8] for the complex remained on average as a predicted structure over 20 ns. Despite the occasional loss and regain of residues, the average number of residues in the α-helices was in agreement with the predicted model of LAT1 (**Figure S9**, **S10**). The time evolution of phi (ϕ) and psi (ψ) dihedral was computed for 15 active site residues that displayed interactions in the docking poses (**Figure S11**). A significant change was observed in the distribution of dihedrals of residues A253, G255, G256, and N258. A large population of dihedrals of these residues was found concentrated in the bottom right corner (or disallowed regions) indicating steric clashes. The high amplitude fluctuations of dihedrals of glycines can be attributed to the lack of Cβ that allows a larger number of combinations of ϕ and ψ. Since these residues are located on the TM6 helix break, high flexibility of this region may be an additional factor behind the drastic fluctuations of dihedrals. No significant deviations were observed in the distribution of dihedral angles of other active site residues. The binding conformation of **31** was very stable throughout the entire simulation with an average RMSD value of 0.28 nm **(Figure S7B)** and with all the described active site interactions completely conserved.

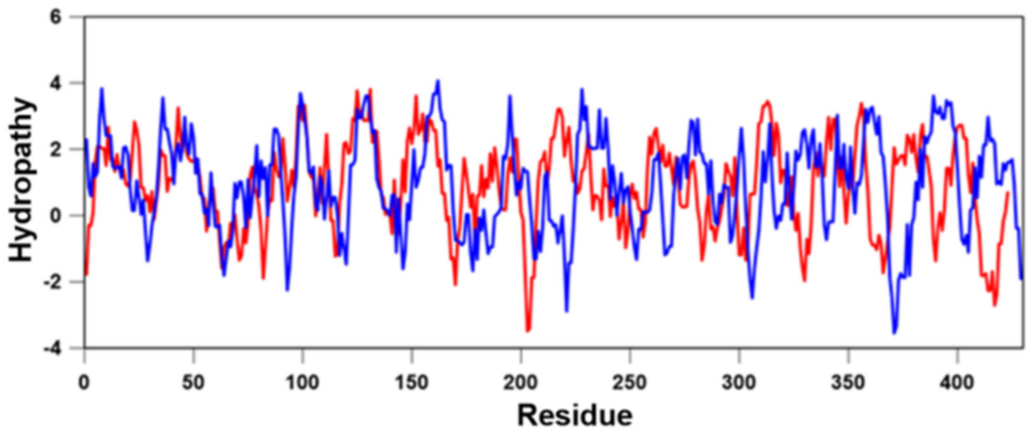

Figure S1. Kyte-Doolittle hydropathy plot of the LAT1 model (Blue) and AdiC (Red).

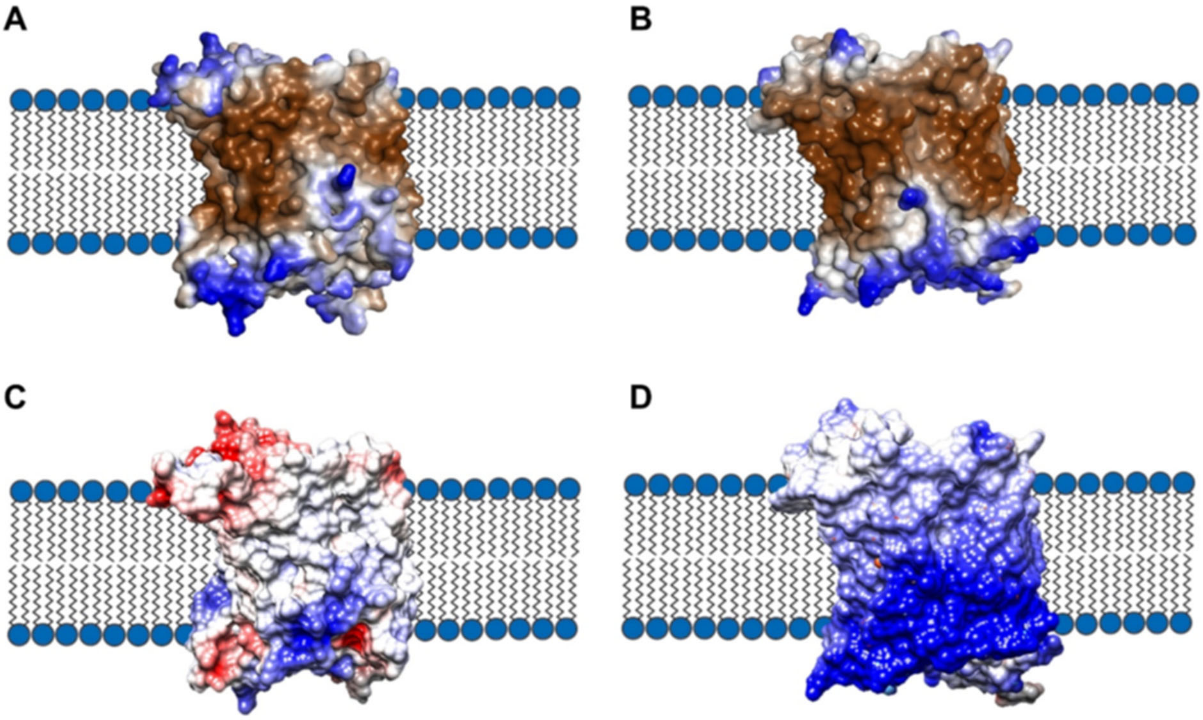

Figure S2. The hydrophobic-hydrophilic surface of LAT1 model (**A**) and AdiC (PDB ID: 3L1L) (**B**). The blue regions correspond to the hydrophilic residues, while the brown areas correspond to the hydrophobic residues. **C** and **D** show the electrostatic potential molecular surface of LAT1 and AdiC, respectively. The electrostatic potential values on the surface range from negative -5 kbT/ec (red) to 5 kbT/ec (blue).

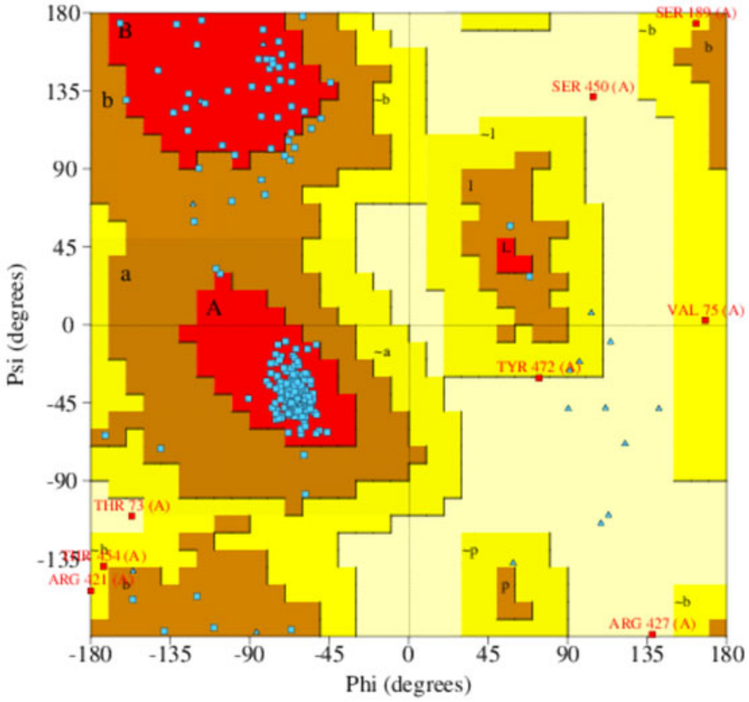

Figure S3. The Ramachandran plot of LAT1 model based on the AdiC structure.

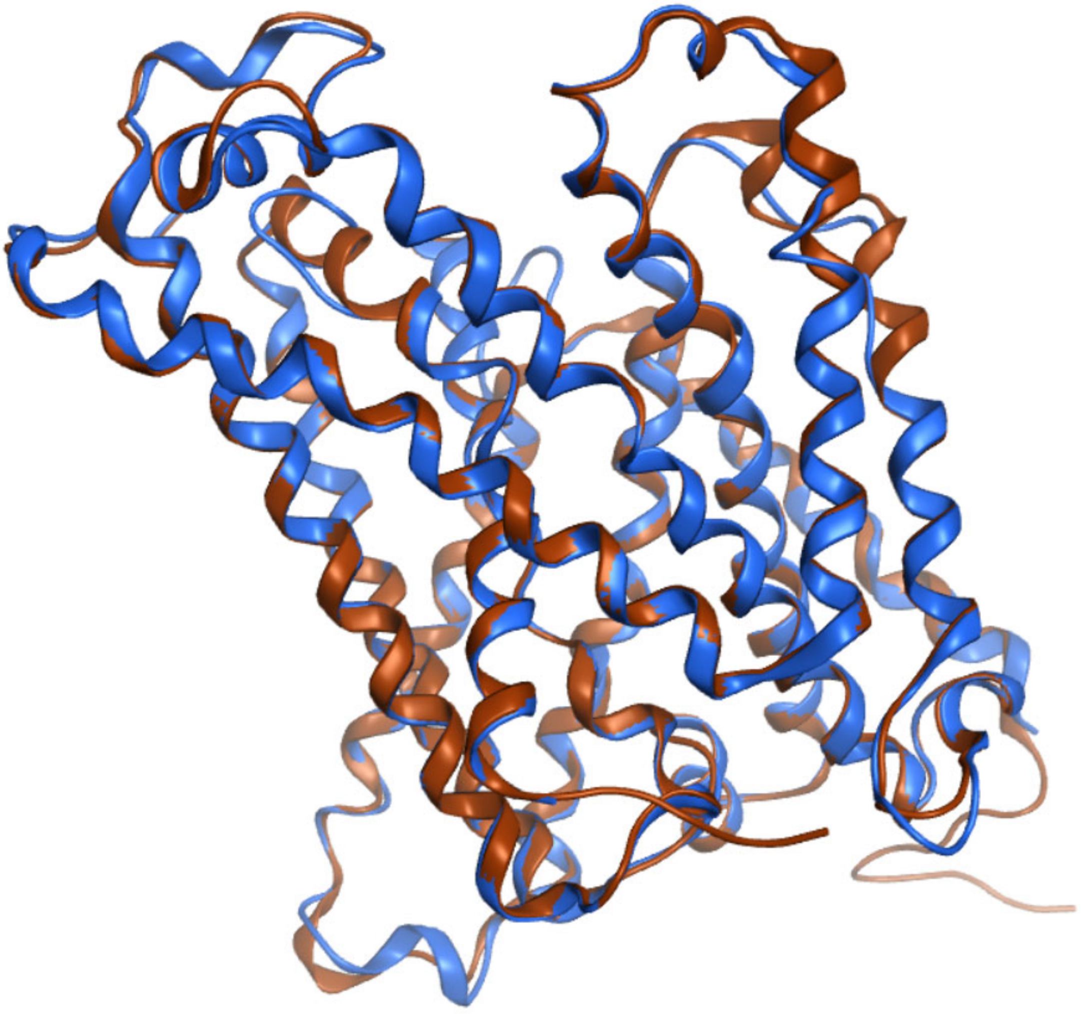

Figure S4. Superimposition of the LAT1 model (blue) and the template x-ray crystal structure of the AdiC (brown, PDB ID: 3L1L). Alignment score: 0.032, Backbone RMSD: 0.62Å.

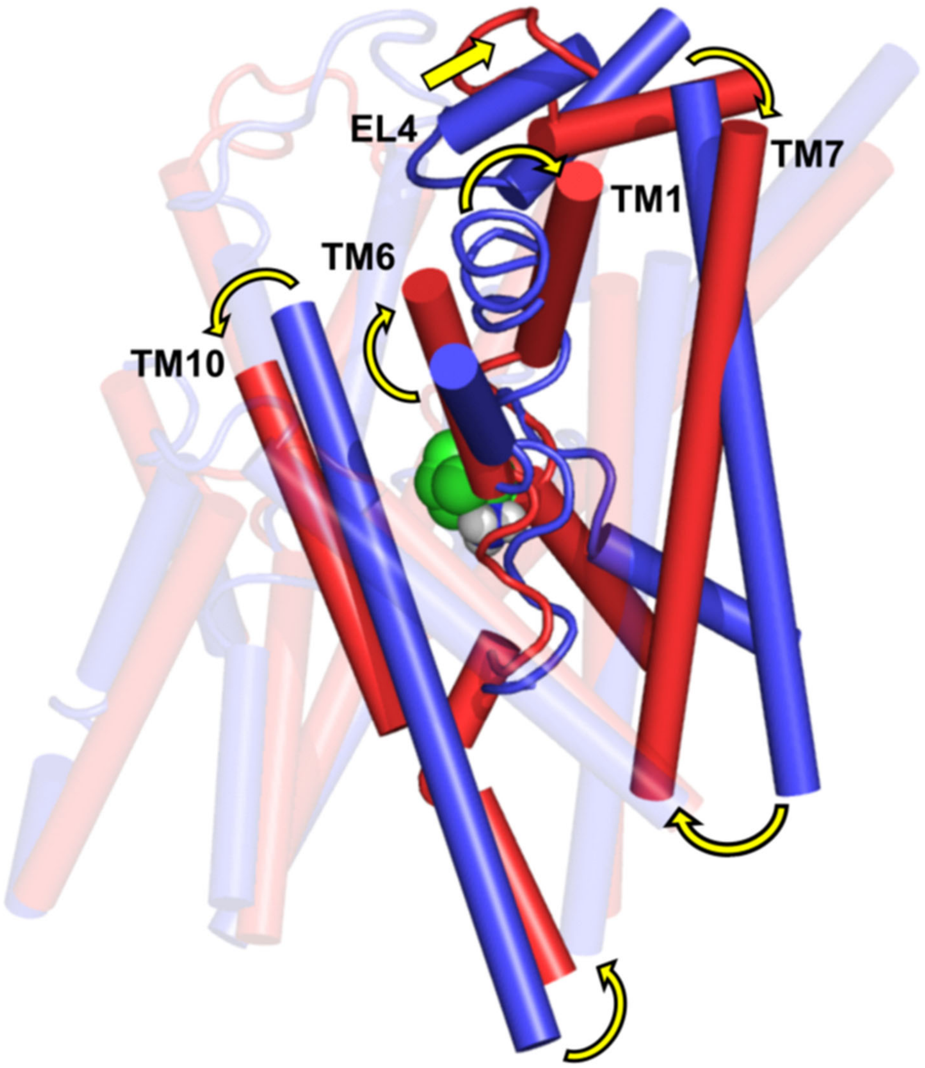

Figure S5. Possible conformational changes of LAT1 involved in the transition from the inward-open state to the outward-open occluded state. To illustrate the differences between the inward-facing structure of LAT1 (blue, PDB ID: 6IRT), bound to BCH (green, space-filling style), and the outward-open occluded model of LAT1 (red), both structures were superpositioned and are represented as cartoon ribbon (helices shown as solid cylinders). Most distinct movements in this conformational change (indicated by yellow arrows) are within transmembrane regions, i.e., transmembrane helices (TMs) 1, 6 and 7. A significant movement was also observed on the periplasmic side in EL4; moderate movement was observed in TM10, and other TMs showed only small-scale changes.

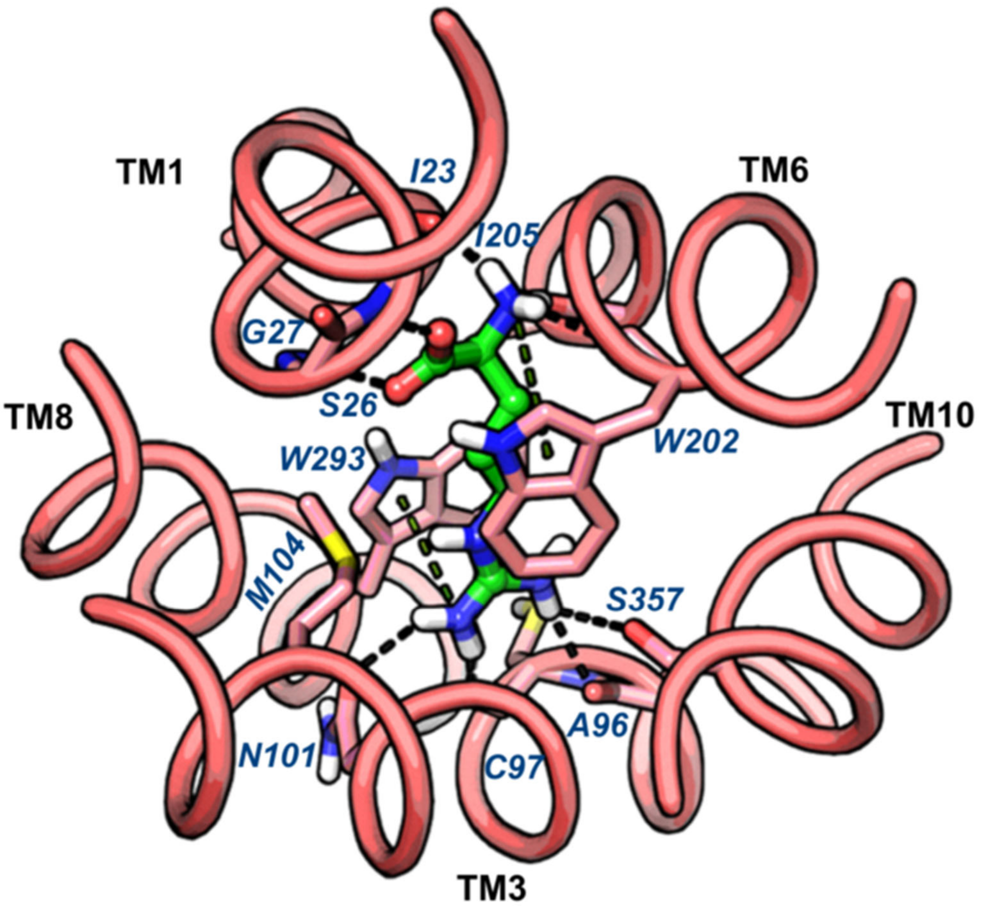

Figure S6. Periplasmic view of the substrate-binding site of AdiC (PDB ID: 3L1L). Arginine (green) is bound to AdiC at the center of the transport path, recognized by amino acids from TM1, TM3, TM6, TM8, and TM10. The amino acid moiety of the arginine is engaged in five backbone hydrogen bond interactions involving residues I23, S26, and G27 in TM1 and W202 and I205 in TM6. S26 and G27 are donating two hydrogen bonds to the α-carboxyl group, and the α-amino group is contributing three hydrogen bonds to the backbone oxygen of I23, W202 and I205. In addition, the positively charged α-amino group is involved in an ionic interaction (cation-π) with the side chain of W202. Furthermore, the guanidinium group at the other end of the arginine is stacking against W293 in TM8 through a cation-π interaction and its nitrogen atoms are donating hydrogen bonds to A96, C97, N101 in TM3, and S357 in TM10. Besides the polar interactions, the aliphatic side chain of the arginine is involved in favorable hydrophobic interactions involving M104 in TM3, and W202 and I205. Arginine and interacting residues of AdiC are shown in stick-ball and stick representation, respectively.

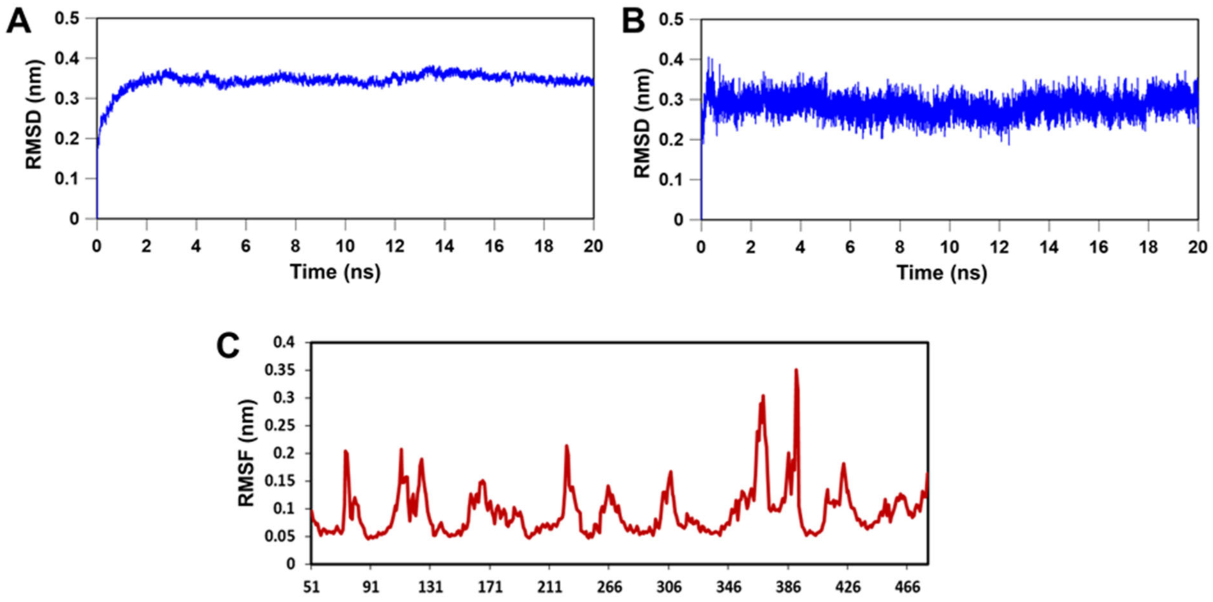

Figure S7. The RMSD plots of the Cα atoms (**A**) and the ligand (**B**) as a function of time for the LAT1-**31** complex. **C**, RMSF of whole protein calculated for last 10 ns time interval.

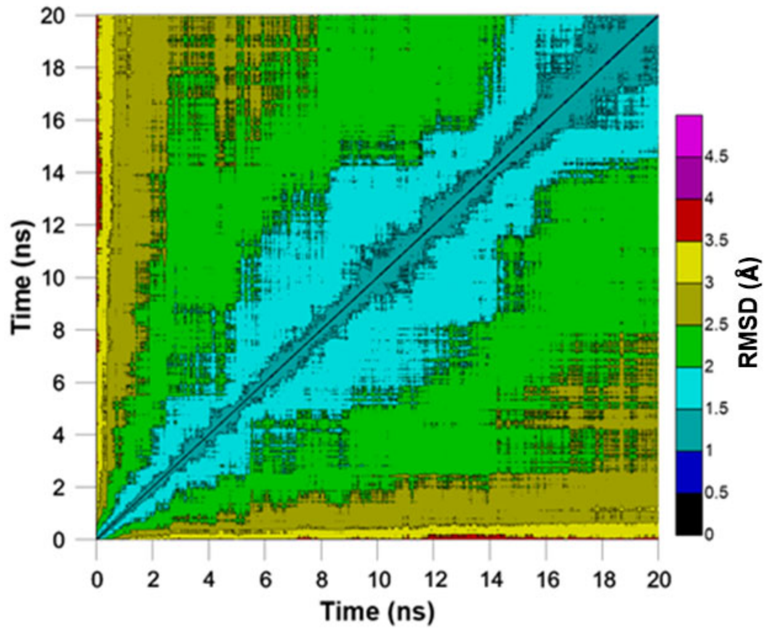

Figure S8. The pairwise Cα RMSD plot generated from the MD simulation of LAT1 complexed with **31**.

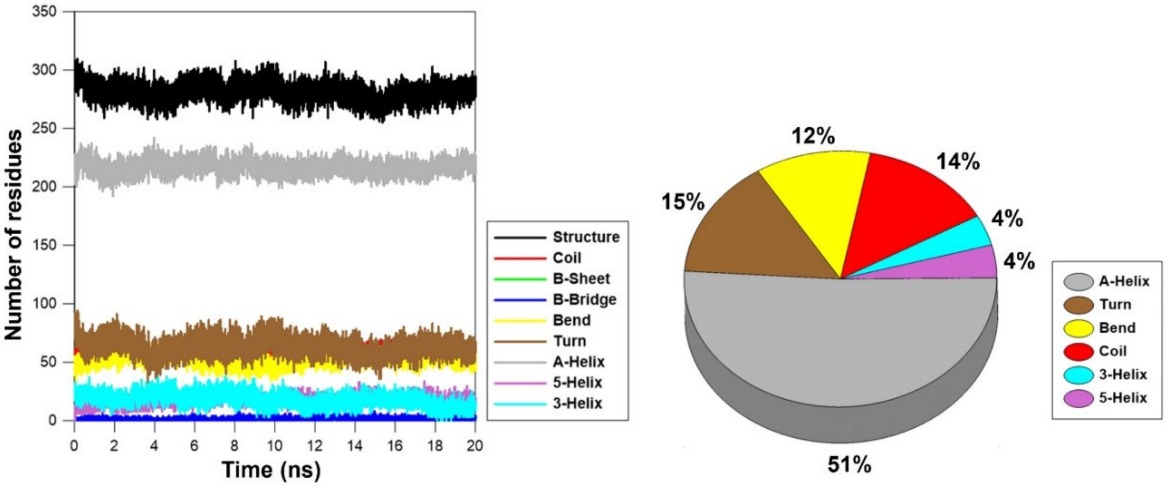

Figure S9. The secondary structure composition of LAT1 bound to **31** during the MD simulation.

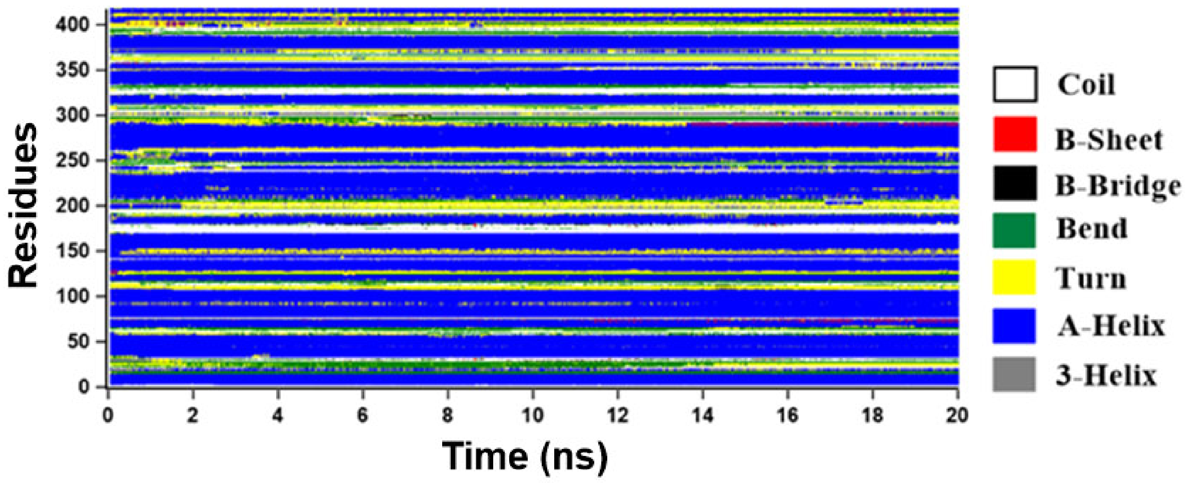

Figure S10. The conformational evolution of the secondary structure elements of LAT1 bound to **31** during the MD simulation.

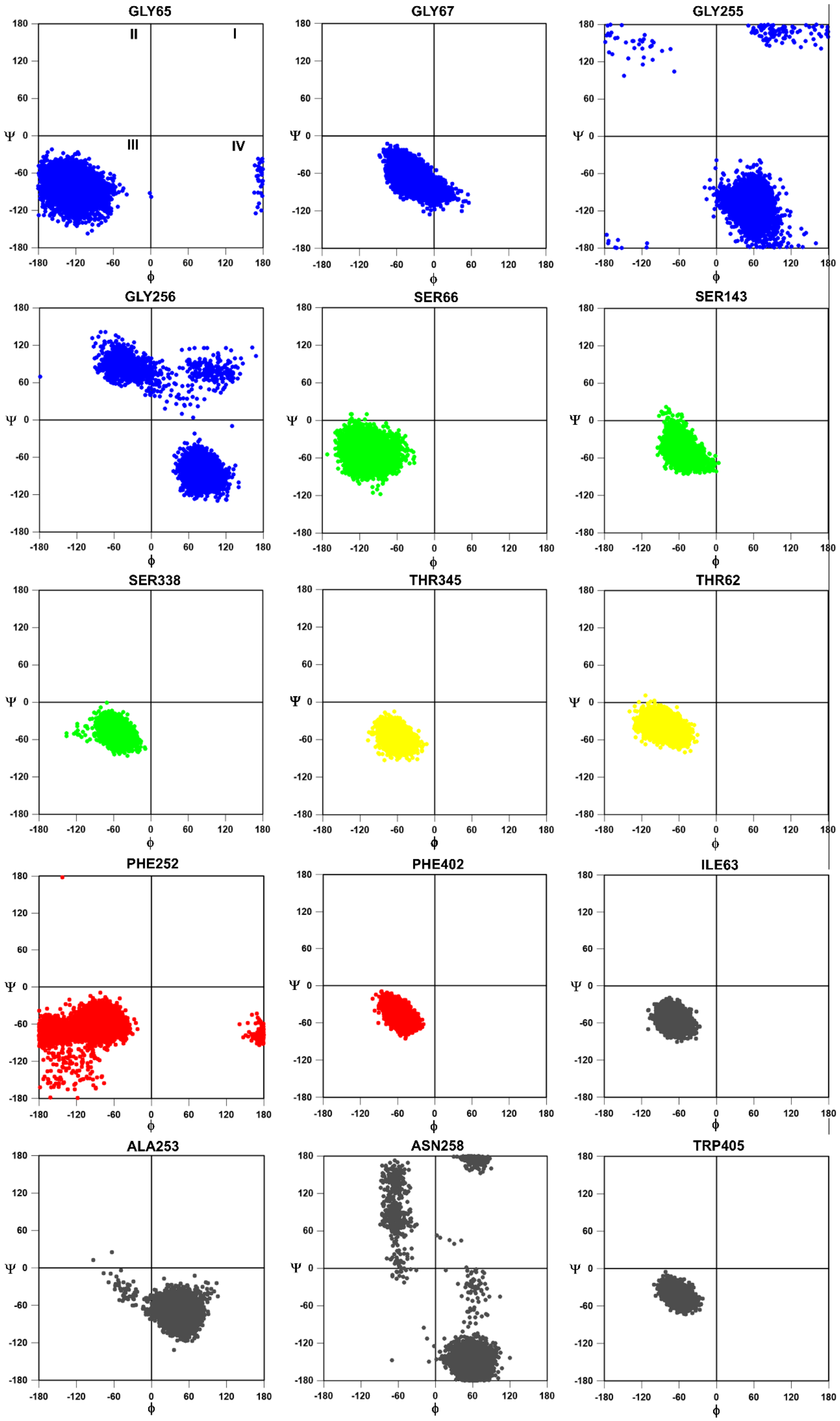
GLY65
GLY67
GLY255
GLY256
SER66
SER143
SER338
THR345
THR62
PHE252
PHE402
ILE63
ALA253
ASN258
TRP405
I
II
III
IV
Ψ
ϕ

Figure S11. The Ramachandran (ϕ–ψ) plots of 15 active site residues obtained from the MD simulation of LAT1 bound to **31**. (Dihedrals of G65, G67, G255, and G256 are represented in blue; S66, S143, and S338 are depicted in green; T345 and T62 are depicted in yellow; F252 and F402 are depicted in red, and I63, A253, N258, and W405 are depicted in dark grey)

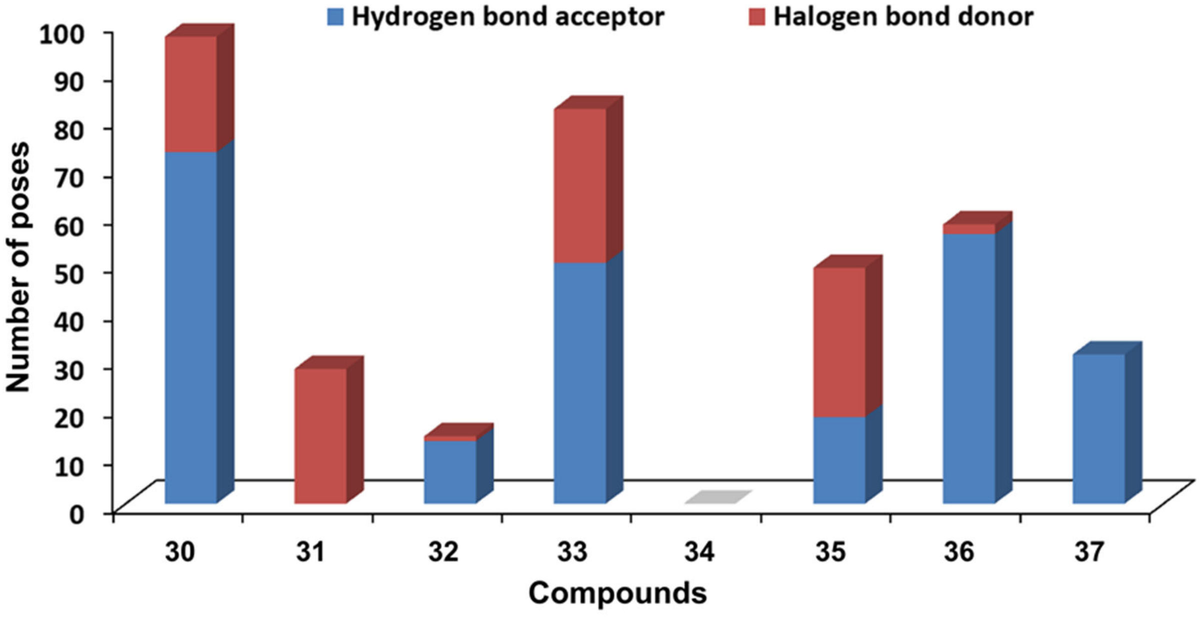

Figure S12. The SIFt analysis showing the halogen interactions in the docking poses of compounds **30**-**37**. The blue vertical bars indicate the poses where the halogen atom is acting as a hydrogen bond acceptor from the residue, and the red vertical bars indicate the poses where the halogen atom is donating an X-bond to the residue. In 236 poses halogen atoms were acting as a hydrogen bond acceptor from the residues S66, G67, S143, Q197, N258, S338, T345 and S342, and in 124 poses halogen atoms were X-bond donor to the backbone oxygen of E136, F252, S338, and S401.

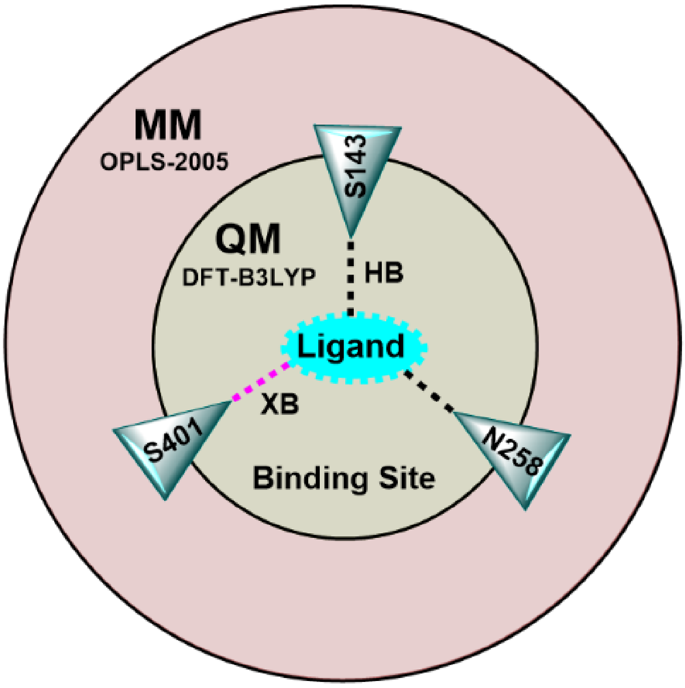

Figure S13. The illustration of the different sections of QM-MM system for geometry optimization (XB: Halogen bond, HB: Hydrogen bond).

Cpd.	pIC_{50}	μ (Debye)	Glide (Extra Precision)				QM-MM					$RMSD_{Ligand}$	ΔΔG (Kcal/mol)
			∠C=O…X<br>∠O-H…X<br>∠N-H…X	d(X…O)<br>d(X…H)	∠C-X…O<br>∠C-X…H	ΔG_{Glide} (Kcal/mol)	∠C=O…X<br>∠O-H…X<br>∠N-H…X	d(X…O)<br>d(X…H)	∠C-X…O<br>∠C-X…H	$E_{QM\text{-}MM}$ (hartree)	$\Delta G_{QM\text{-}MM}$ (Kcal/mol)		
**30**	-	10.30	114.9 (O)	3.00 (O)	156.8 (O)	-20.56	101.6 (O)	3.33 (O)	151.7 (O)	-1151.75	-51.60	0.11	-31.03
**31**	5.10 [9]	10.36	114.9 (O)	3.01 (O)	152.6 (O)	-39.92	127.9 (O)	3.39 (O)	164.3 (O)	-1162.07	-68.03	0.26	-28.10
**32**	-	11.58	152.0 (H)	2.7 (H)	104.2 (H)	-29.32	145.5 (O)	2.49 (H)	91.9 (H)	-742.87	-54.28	0.16	-24.96
**33**	-	9.12	102.1 (O)	3.42 (O)	150.7 (O)	-20.49	99.0 (O)	3.23 (O)	156.9 (O)	-1117.54	-39.32	0.38	-18.83
**34**	4.57 [10]	9.96	-	-	-	-5.45	-	-	-	-790.89	-17.78	0.20	-12.32
**35**	-	9.56	92.8 (O)	3.26 (O)	164.5 (O)	-24.74	100.7 (O)	3.23 (O)	170.8 (O)	-1525.24	-48.87	0.75	-24.12
**36**	5.67 [11]	9.74	104.0 (O)	2.91 (O)	162.4 (O)	-39.68	99.1 (O)	3.93 (O)	128.3 (O)	-2314.15	-64.14	0.57	-24.46
**37**	5.30 [11]	10.60	91.7 (O)<br>152.7 (H)	3.23 (O)<br>3.45 (H)	165.5 (O)<br>103.7 (H)	-41.82	105.2 (O)<br>128.6 (H)	3.22 (O)<br>2.67 (H)	132.27 (O)<br>114.2 (H)	-2442.56	-61.95	0.70	-20.12
**BCH**	-	-	-	-	-	-35.95	-	-	-	-540.77	-27.20	1.46	8.75
**L-phe**	-	-	-	-	-	-10.04	-	-	-	-577.70	-33.78	0.59	-23.74

Table S1. PIC_{50} : Biological activity of the ligand; μ: Dipole moment of the ligand; ∠C=O…X represents the acceptor angle in degrees where (O) is an X-bond acceptor; ∠O-H…X, ∠N-H…X represents the donor angle where X is a hydrogen bond acceptor; d(X…O), d(X…H) represents the distance in angstrom (Å) between X and (O) or (H); ∠C-X…O represents the donor angle or σ-hole angle where X is an X-bond donor; ∠C-X…H represents the acceptor angle where X is a hydrogen bond acceptor; ΔG_{Glide} represents the binding energy (MM-GBSA) of the docking pose obtained from the Glide XP docking; $\Delta G_{QM\text{-}MM}$ represents the binding energy (MM-GBSA) of the QM-MM optimized docking pose; $E_{QM\text{-}MM}$ represents the QM-MM optimization energy; $RMSD_{Ligand}$ represents the root mean square deviation of the ligand after QM-MM optimization; ΔΔG represents the improvement in binding energy of the ligand after QM-MM optimization.

Cpd.	X	Q_X	ΔE (kcal mol^{-1})	$V_{S,\,max}$ (kcal mol^{-1})
**30**	I	0.081	-2.69	38.44
**31**	I	0.077	-4.96	42.79
**33**	Br	-0.034	-2.33	20.73
**35**	Cl	-0.102	-1.85	11.93
**36**	Cl	-0.148	-0.85	4.37
**37**	Cl	-0.139	-0.99	4.79

Table S2. Q_X: Electrostatic charge of the halogen atom; ΔE: Interaction energy of the ligand with S401; $V_{S,max}$: Maximum positive potential on the halogen atom.

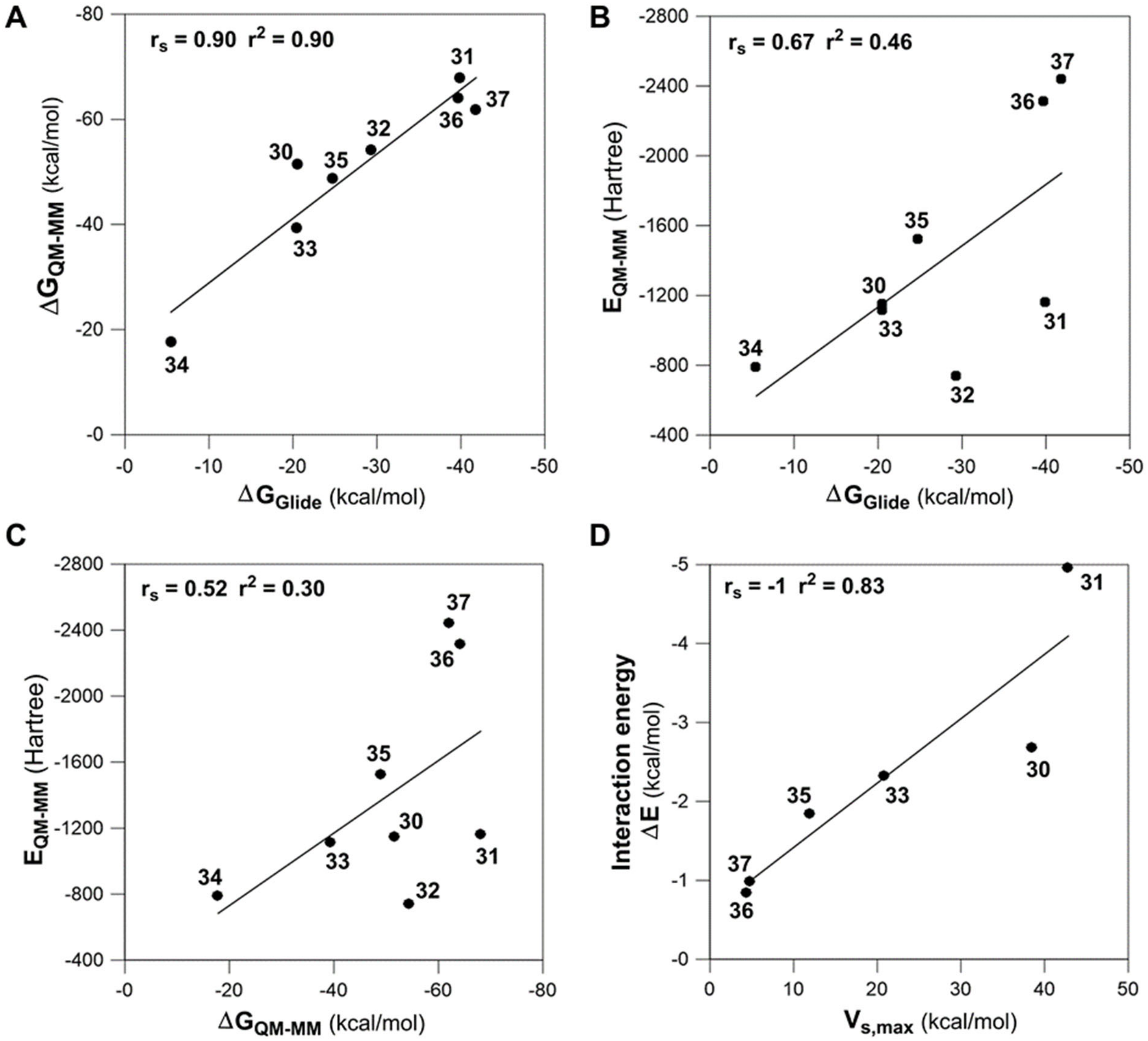

Figure S14. Plots of the computed energy values and their respective Spearman's rank correlation coefficient (**r_s**) and regression coefficient (**r^2**). **A**, $\Delta G_{QM\text{-}MM}$ vs. ΔG_{Glide} (Correlation is significant, $p < 0.01$) **B**, QM-MM energy vs. ΔG_{Glide} (No statistically significant difference in means is observed, $p = 0.071$) **C**, QM-MM energy vs. $\Delta G_{QM\text{-}MM}$ (No statistically significant difference in means is observed, $p = 0.183$) **D**, Interaction Energy (ΔE) vs. $V_{S,max}$ (Correlation is significant, $p < 0.01$).

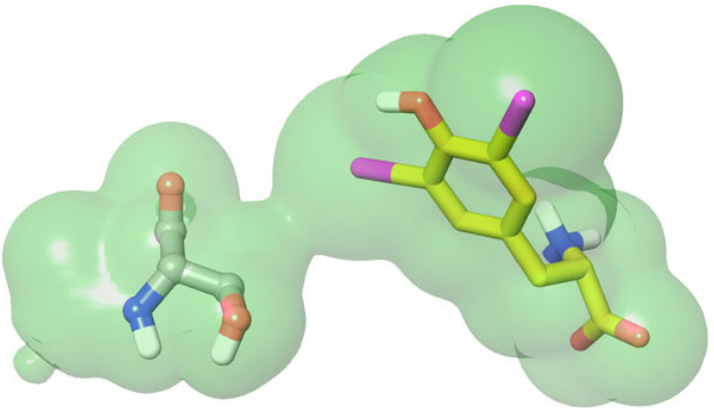

Figure S15. QM-MM optimized pose of a random docking pose of **31** showing the absence of a bond critical point between the iodine and backbone oxygen atom of S401. The ligand and residue are shown in stick-ball and stick style, respectively. $\Delta G_{QM\text{-}MM}$ = -48.04 kcal mol^{-1}

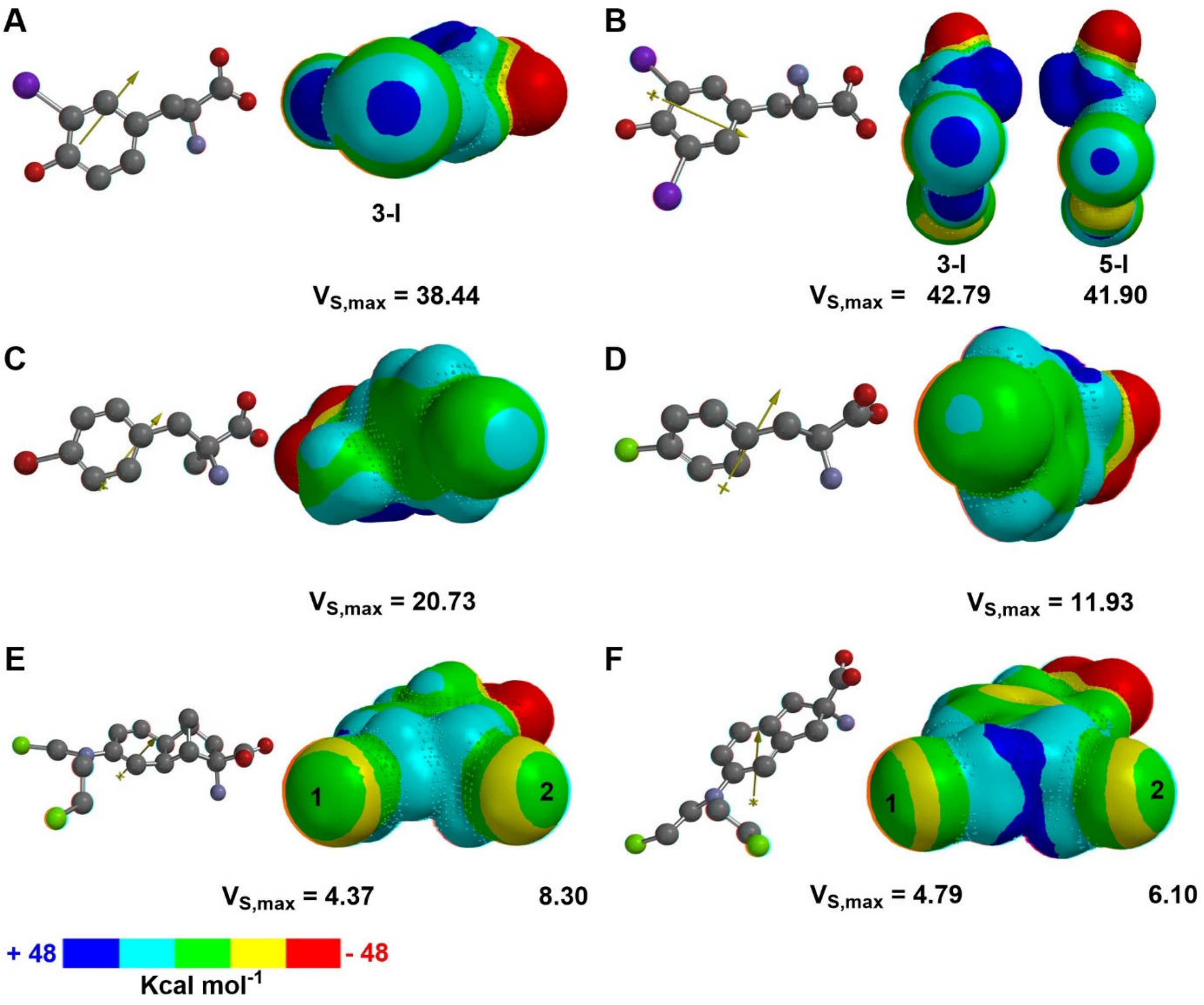

Figure S16. The electrostatic potentials mapped onto the molecular surface of **30** (**A**), **31** (**B**), **33** (**C**), **35** (**D**), **36** (**E**), and **37** (**F**). The arrow over the ligand (shown in stick-ball representation in the left) indicates the dipole moment vector.

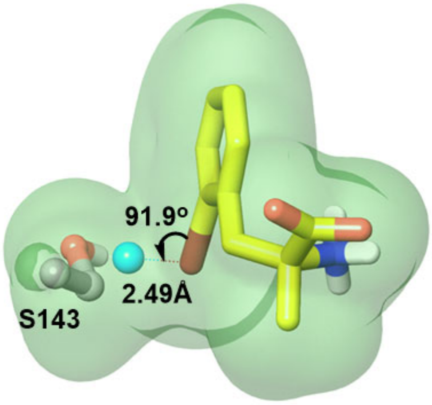

Figure S17. QM-MM optimized pose of **32**.

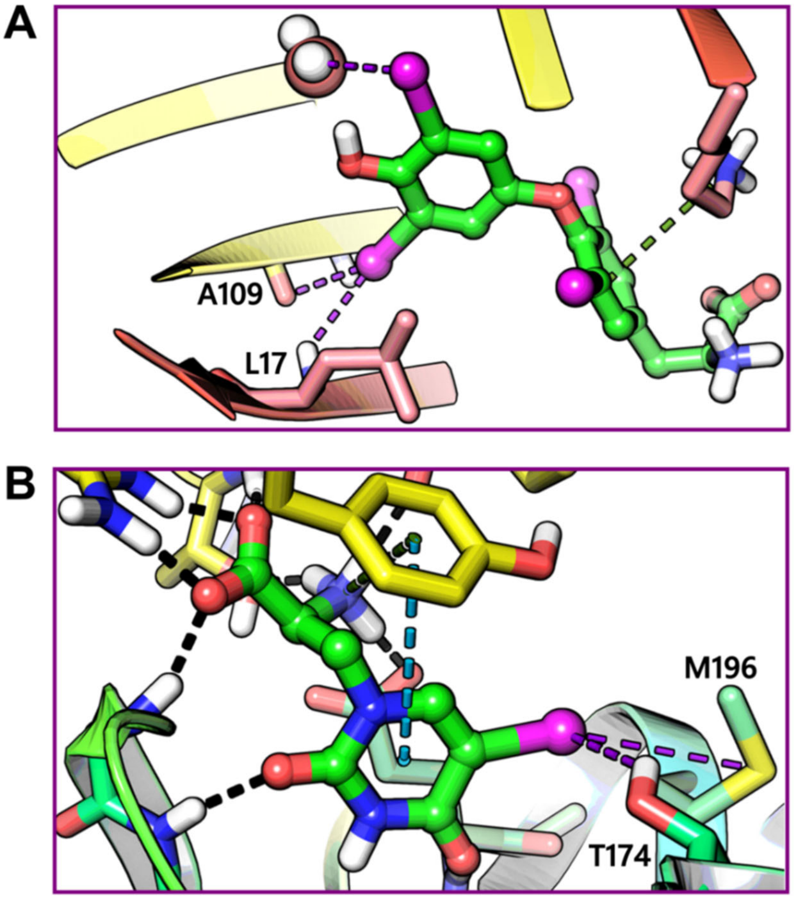

Figure S18. A, Binding mode of T_4 (green, ball-stick) in Transthyretin (PDB ID: 1IE4). The 3′ iodine is involved in a hydrogen bond interaction with a nearby water molecule (depicted in space-filling style), while the 5′ iodine is engaged in backbone interactions involving A109 and L17. **B,** Binding mode of 5-iodowillardiine (green, ball-stick) to glutamate receptor (PDB ID: 1MY4). The iodine atom is involved in two polar contacts, hydrogen bond and halogen bond, with the residues T174 and M196. The halogen interactions in **A** and **B** are indicated as violet dotted lines.

References

1. Kyte, J. & Doolittle, R. F. A simple method for displaying the hydropathic character of a protein. *J. Mol. Biol.* **157**, 105–132 (1982).
2. Baker, N. A., Sept, D., Joseph, S., Holst, M. J. & McCammon, J. A. Electrostatics of nanosystems: application to microtubules and the ribosome. *Proc. Natl. Acad. Sci. U.S.A.* **98**, 10037–10041 (2001).
3. Dolinsky, T. J., Nielsen, J. E., McCammon, J. A. & Baker, N. A. PDB2PQR: an automated pipeline for the setup of Poisson-Boltzmann electrostatics calculations. *Nucleic Acids Res.* **32**, W665-667 (2004).
4. Dolinsky, T. J. *et al.* PDB2PQR: expanding and upgrading automated preparation of biomolecular structures for molecular simulations. *Nucleic Acids Res* **35**, W522–W525 (2007).
5. Sorin, E. J. & Pande, V. S. Exploring the Helix-Coil Transition via All-Atom Equilibrium Ensemble Simulations. *Biophys J* **88**, 2472–2493 (2005).
6. Li, H., Robertson, A. D. & Jensen, J. H. Very fast empirical prediction and rationalization of protein pKa values. *Proteins* **61**, 704–721 (2005).
7. Kabsch, W. & Sander, C. Dictionary of protein secondary structure: pattern recognition of hydrogen-bonded and geometrical features. *Biopolymers* **22**, 2577–2637 (1983).
8. Touw, W. G. *et al.* A series of PDB-related databanks for everyday needs. *Nucleic Acids Res.* **43**, D364-368 (2015).
9. Geier, E. G. *et al.* Structure-based ligand discovery for the Large-neutral Amino Acid Transporter 1, LAT-1. *Proc. Natl. Acad. Sci. U.S.A.* **110**, 5480–5485 (2013).
10. Wiriyasermkul, P. *et al.* Transport of 3-fluoro-L-α-methyl-tyrosine by tumor-upregulated L-type amino acid transporter 1: a cause of the tumor uptake in PET. *J. Nucl. Med.* **53**, 1253–1261 (2012).
11. Matharu, J., Oki, J., Worthen, D. R., Smith, Q. R. & Crooks, P. A. Regiospecific and conformationally restrained analogs of melphalan and DL-2-NAM-7 and their affinities for the large neutral amino acid transporter (system LAT1) of the blood-brain barrier. *Bioorg. Med. Chem. Lett.* **20**, 3688–3691 (2010).
